# Environmental Filtering Weakens with Trophic Level in Urban Coastal Ecosystems

**DOI:** 10.1021/acs.est.5c08142

**Published:** 2026-01-30

**Authors:** Wenqian Xu, Yu-De Pei, Taylor M. W. Li, Joshua Bennett-Williams, Ruixian Sun, Shara K. K. Leung, Masayuki Ushio, Alex S. J. Wyatt, Charmaine C. M. Yung

**Affiliations:** Department of Ocean Science, 58207 The Hong Kong University of Science and Technology, Hong Kong SAR 999077. China

**Keywords:** coastal coral ecosystem, community diversity, environmental DNA, multitrophic interaction

## Abstract

Urban coastal ecosystems face increasing anthropogenic pressures and environmental variability, yet the consequences for multitrophic biodiversity and ecosystem networks remain poorly resolved. Here, we combine environmental DNA metabarcoding, visual surveys, flow cytometry, and environmental measurements to examine the spatiotemporal dynamics of marine metazoans, protists, and prokaryotes across estuarine, transitional, and oceanic habitats in Hong Kong’s urbanized coastal waters. Using permutational multivariate analysis of variance (PERMANOVA), we demonstrate that environmental control over community composition weakens systematically at higher trophic levels. The variance explained by seasonal and spatial interaction was highest for prokaryotes (*R* ^2^ = 0.76) and protists (0.59), but notably lower for benthic fauna (0.41) and bony fish (0.32). Co-occurrence network analysis revealed that oceanic habitats, dominated by heterotrophic prokaryotes, omnivorous fish, and hard corals, supported the most complex and stable multitrophic networks, with an average complexity of 0.54 compared to estuarine (0.23) and transitional habitats (0.29). Structural equation modeling further revealed habitat-specific drivers: temperature exerted the strongest direct effect in estuarine habitats (>0.44), while biotic interactions involving primary producers played a dominant role in oceanic habitats (direct effect >0.28). In contrast, transitional habitats lacked significant environmental or biotic drivers, indicating a system in flux where community dynamics are likely governed by complex variables beyond standard environmental or biotic regulation. These findings demonstrate the gradient-dependent interplay of environmental filtering and biotic regulation in shaping coastal ecosystem stability. Our results also highlight the value of an integrated eDNA-based framework for monitoring biodiversity and ecosystem change, providing insights for the management of urban marine environments under global change.

## Graphical abstract

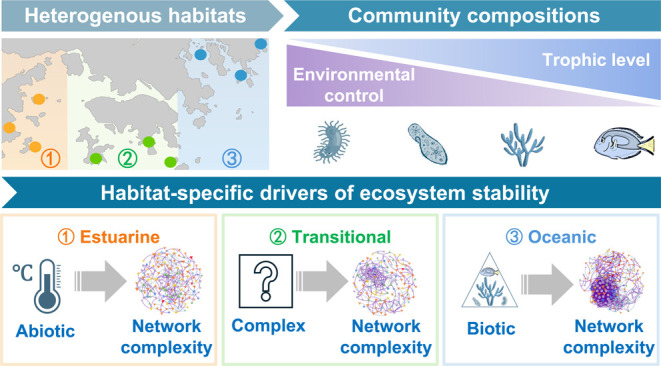

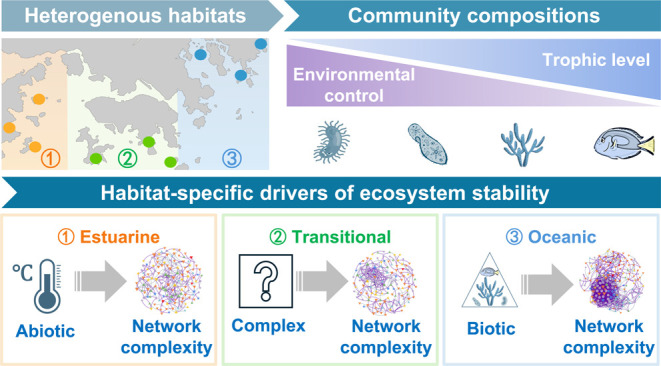

## Introduction

Urban coastal ecosystems near rapidly expanding cities are increasingly subject to combined pressures from localized pollution and global climate change.^1,2^ Urban coastal waters thus serve as critical ecological frontiers for understanding how natural communities respond and reorganize under intense human influence. While it is well established that these stressors shift community distributions and compositions,^3–5^ a major uncertainty remains regarding how they impact the stability of multitrophic network architecture. This architecture represents the complex web of interactions that sustains ecosystem function. Environmental stressors can fundamentally reshape the flow of energy and matter through aquatic food webs and potentially alter ecosystem function and resilience.^6^ In this context, ecosystems function as complex webs defined by the intricate predator–prey, competitive, and symbiotic interactions that link organisms together.^7,8^ Consequently, the persistence of these systems depends on ecological stability, defined as the ability to maintain structural and functional integrity, and its key component, resilience, which measures the capacity for rapid recovery following a disturbance. Determining whether environmental forcing selectively decouples these trophic linkages or reorganizes the entire interaction web is therefore essential for predicting ecosystem resilience under intense human influence.

Bridging this gap requires a tool capable of simultaneously surveying biodiversity from microbes to macro-organisms. Traditional methods, such as trawling or visual censuses, are often taxonomically restricted and logistically constrained in turbid, high-traffic urban waters where visibility is poor.^9,10^ Environmental DNA (eDNA) metabarcoding overcomes these limitations by detecting genetic material shed into the environment.^11,12^ This approach enables the rapid, noninvasive, and comprehensive assessment of biodiversity across the entire trophic spectrum in a single sample, providing the taxonomic resolution necessary to reconstruct complex ecological networks.^13^

However, the application of eDNA has largely focused on descriptive biodiversity inventories or distribution mapping,^14–16^ often neglecting the ecological mechanisms that structure these communities.^17–19^ A key barrier to predicting ecosystem resilience is understanding how environmental control varies across trophic levels. Metabolic theory suggests that basal organisms with rapid generation times, such as microbes, are tightly coupled to physical parameters like temperature.^20,21^ Conversely, higher trophic levels may be buffered against direct environmental forcing by mobility and behavioral adaptations.^22^ If environmental filtering acts asymmetrically across trophic levels,^23^ climate anomalies could desynchronize the food web, leading to a breakdown in energy transfer and ecosystem stability.^24^ In this scenario, ecosystem resilience may critically depend on “keystone taxa”, which are connector species that bridge the fast-responding microbial loop and higher-order metazoan consumers.^25,26^ By integrating energy flow and coupling top-down with bottom-up controls, these taxa help buffer the system against perturbations and can serve as indicators of resilience.^27–29^

While eDNA-based network analysis is a promising frontier,^29,30^ pioneering studies have often relied solely on co-occurrence patterns. These patterns may reflect shared environmental preferences rather than true biological interactions and frequently lack seasonal resolution or validation against established methods. Bridging this gap between biodiversity patterns and ecological processes requires integrated, multimethod approaches. Combining the broad taxonomic coverage of eDNA with established techniques, such as underwater visual censuses for fish and coral community and flow cytometry for microbial populations, enables robust insights into community dynamics and network structure across spatial and temporal scales. The subtropical coastal waters of Hong Kong provide an exemplary system to test these ideas. The region exhibits a pronounced west-to-east gradient driven by massive freshwater and nutrient inputs from the Pearl River Estuary contrasted against oceanic influences from the South China Sea.^31,32^ This gradient creates distinct estuarine, transitional, and oceanic habitats with varying salinity, turbidity, and pollution levels.^33,34^ Strong seasonal monsoons further modulate water temperature, nutrient availability, and community composition.^35–37^ The interplay between these spatial gradients and temporal fluctuations makes Hong Kong an ideal natural laboratory for disentangling environmental drivers of multitrophic community structure and stability.

This study combines eDNA metabarcoding, underwater visual census, and flow cytometry to investigate the spatial-temporal dynamics of marine communities in Hong Kong coastal waters. We aim to elucidate mechanisms shaping biodiversity and ecological network structure in this heavily urbanized coastal ecosystem. First, we examine how environmental heterogeneity across space and time differentially influences prokaryotes, protists, metazoans, and bony fish. We hypothesize that microbial communities, given their rapid generation times, will respond more strongly to temporal water quality fluctuations, whereas macro-organismal communities will track persistent spatial gradients. Second, we investigate how multitrophic ecological network structure and complexity vary across habitats and seasons, predicting greater network complexity and stability in the environmentally consistent oceanic habitat compared to the fluctuating, stress-prone estuarine environment. Finally, we identify primary environmental drivers of network structure and their implications for ecosystem resilience. We hypothesize that seasonality will dominate network dynamics, and that specific keystone taxa linking microbial and metazoan food webs are critical for maintaining network integrity. By testing these hypotheses, this research advances fundamental understanding of community assembly and resilience in urbanized marine ecosystems while providing scientific foundations for effective, ecosystem-based management strategies.

## Results

### Validation of eDNA Metabarcoding Through Complementary Survey Methods

We surveyed biodiversity across three distinct Hong Kong marine habitats in 2023 (Figure 1a): estuarine, transitional, and oceanic. We collected 108 benthic water samples quarterly at nine sites. For robust comparative analyses, sequence data were rarefied to consistent depths, excluding one sample (PC2_Feb) due to insufficient sequences. The final data sets consisted of 18,557 reads for prokaryotes (402 ASVs), 33,936 for protists (357 ASVs), 34,046 for benthic fauna (557 ASVs), and 48,271 for bony fish (159 molecular OTUs at 98.5% identity). Rarefaction curves (Figure S1) reached asymptotes for all groups, confirming that the sequencing depth was sufficient to capture the majority of diversity within the samples.

**Figure 1:**
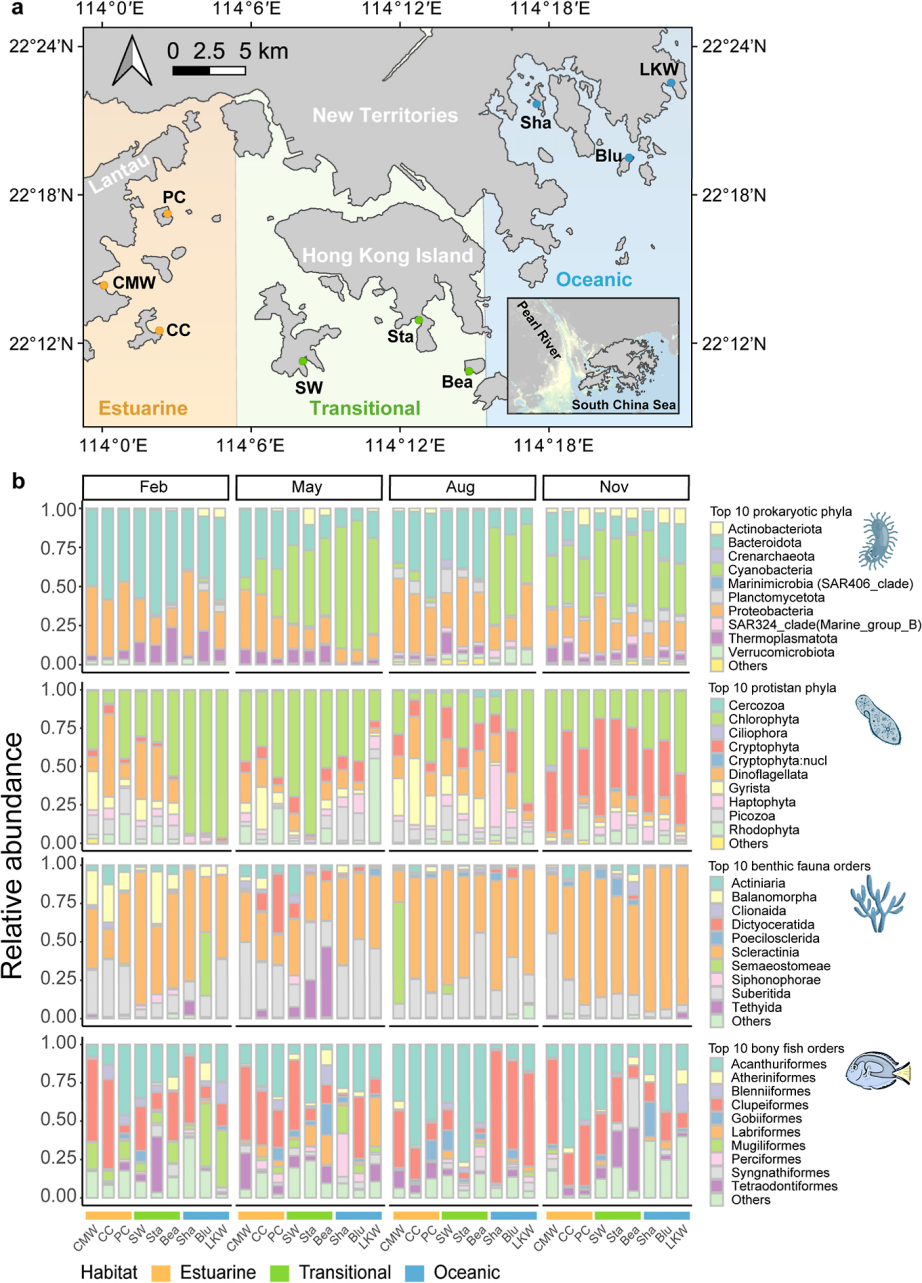
Sampling design and taxonomic composition across Hong Kong waters. (a) Map showing the study area with sampling sites distributed across three distinct ecological habitats: estuarine (orange) in the western waters including CMW, CC, and PC sites; transitional (green) around Hong Kong Island including SW, Sta, and Bea sites; and Oceanic (blue) in the eastern waters including LKW, Sha, and Blu sites. Inset shows the location of Hong Kong relative to mainland China and the South China Sea, with the Pearl River Estuary highlighted in yellow. (b) Bar plots showing the relative abundance of top 10 taxonomic groups for four major categories across the three habitats during four seasonal sampling periods (February, May, August, November). From top to bottom: prokaryotic phyla, protistan phyla, benthic fauna orders, and bony fish orders. Each vertical bar represents a sampling site, with sites organized by habitat (indicated by colored bars at bottom) within each seasonal panel.

To ensure data reliability, we cross-validated eDNA metabarcoding results with traditional visual surveys and flow cytometry (FCM). For benthic communities, while poor water visibility compromised visual photoquadrat surveys (Table S4), both methods identified *Pavona* as the dominant hard coral. Both techniques also showed that oceanic habitats supported higher hard coral cover (10.92 ± 13.33% visual coverage vs 48.81 ± 29.03% eDNA relative abundance; Figure S2). However, notable taxonomic discrepancies emerged between methods. eDNA detected high abundances of *Favites*, *Oulastrea*, and *Cyphastrea* that were rarely observed visually, while showing low abundances of the visually common genus *Acropora*. For fish communities, eDNA demonstrated enhanced detection capability by identifying 30 species compared to only 19 species from visual surveys (using >0.5% relative abundance threshold, Figure S3), with only nine species detected by both methods (Table S14). This advantage was critical at the estuarine Peng Chau site in August, where eDNA successfully characterized fish taxa despite near-zero visibility. Despite these differences, both methods revealed a consistent spatial pattern of higher fish abundance in oceanic habitats (Figure S3).

The quantitative accuracy of the microbial eDNA data was validated against absolute cell counts derived from flow cytometry (FCM). Regression analysis revealed a robust concordance between eDNA sequence reads and FCM data across all microbial groups (*R* ^2^ = 0.52–0.99; Figure S4). This correlation was especially strong for heterotrophic prokaryotes and eukaryotic phytoplankton, where 70.8% of samples showing an *R* ^2^ value above 0.80. These strong correlations confirm that our eDNA relative abundance data provides a reliable proxy for microbial community structure across the sampled environmental gradients.

### Temporal Dynamics of Multiple Organisms Across Habitats

Taxonomic profiling (Figure 1b) and indicator species analyses (Table S6) revealed a clear divergence in spatiotemporal dynamics across trophic levels. Basal organisms exhibited strong seasonal turnover, responding rapidly to water column changes. Prokaryotic communities were driven by a marked summer increase in cyanobacteria (August), while protist communities shifted from winter chlorophyta assemblages to autumn cryptophyte-rich communities. Within this group, dinoflagellates showed bimodal abundance peaks in February and from August to November, especially in estuarine habitats. In contrast, higher trophic levels were structured primarily by spatial gradients rather than seasonal changes. Hard corals (scleractinia) followed a clear spatial gradient, declining from an average of 65.5% in oceanic habitat to 57.3% in transitional and 47.3% in estuarine habitats (Figure 1b). Indicator species analysis reinforced this partitioning, identifying *Pavona* as the indicator taxa for oceanic habitats and *Oulastrea* for transitional habitats (Table S6). Similarly, fish communities exhibited strong spatial specialization despite maintaining a relatively consistent structure year-round. For instance, the herbivorous *Siganus fuscescens* dominated transitional and estuarine habitats in August, whereas the planktivorous *Spratelloides gracilis* prevailed in oceanic habitats.

### Reconstruction of a Coupled Trophic Network

To provide an ecological scaffold for interpreting community associations, we reconstructed a conceptual trophic metaweb for Hong Kong coastal ecosystems using the validated eDNA and survey data, (Figure 2). The network encompasses 17 trophic guilds across four major taxonomic groups, supported by established ecological knowledge. The foundation of this network is built upon a diverse array of primary producers and a rich decomposer community. The primary producer base included autotrophic prokaryotes (4 genera), autotrophic protists (25 genera). And hard corals (19 genera) serve a dual role as both producers (via symbionts) and consumers. Supporting the system’s nutrient recycling capacity, a highly diverse community of heterotrophic prokaryotes (131 genera) constituted the principal decomposer guild.

**Figure 2:**
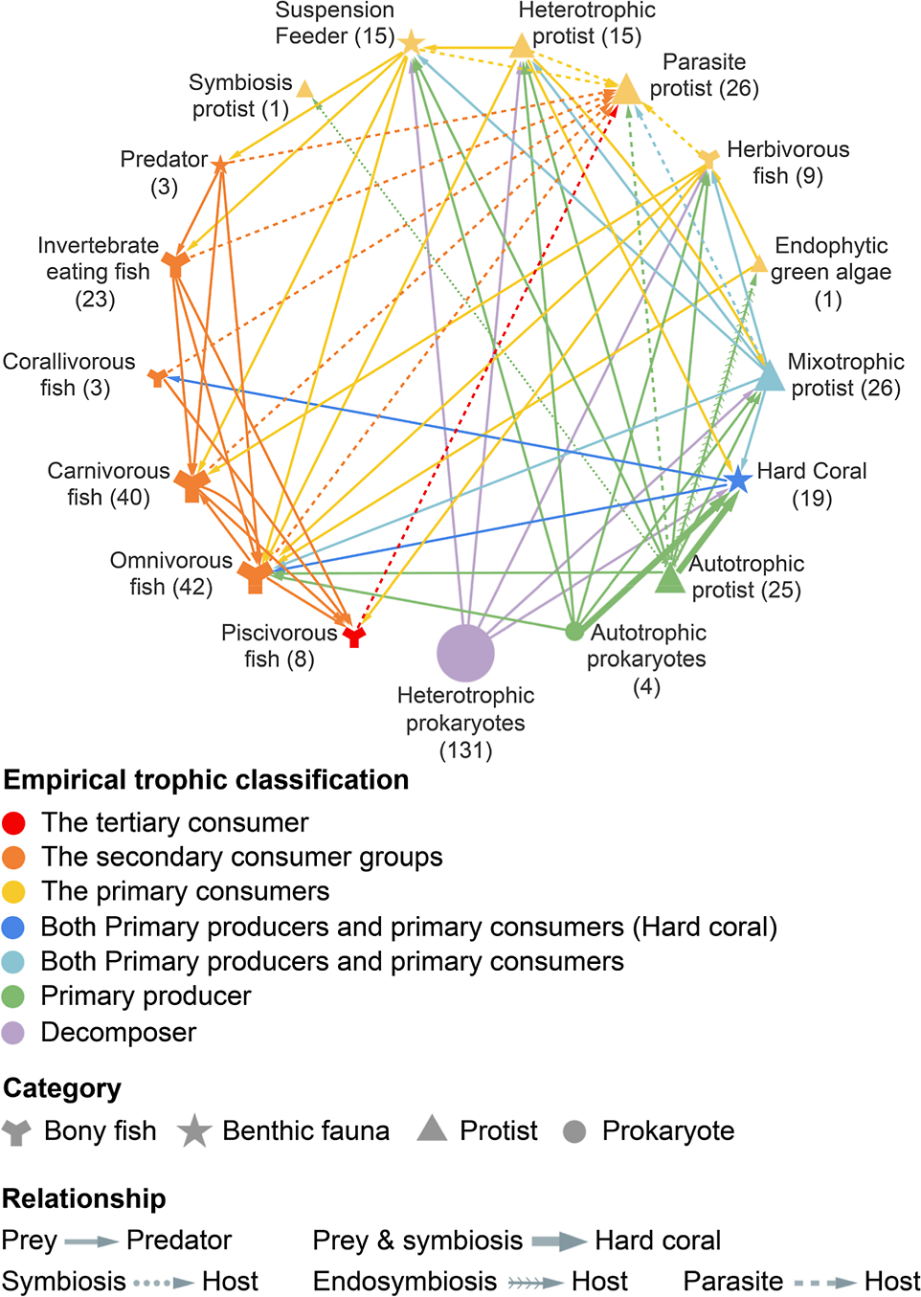
Trophic metaweb reconstructed from detected taxa via eDNA data and established ecological knowledge across Hong Kong coastal ecosystems. Nodes represent different functional groups, with node size proportional to taxonomic richness (values in parentheses). “Suspension Feeder” excludes genera classified as “Hard Coral”. Bony fish richness is calculated at species level, while richness of other groups is calculated at genus level. The metaweb comprises 17 nodes connected by 63 edges, excluding taxonomically unresolved groups.

Energy is channeled from this base upward through several key consumer pathways. Protists, in particular, occupied a critical intermediate position, exhibiting high trophic diversity as heterotrophs (15 genera), mixotrophs (26 genera), and parasites (26 genera). This diversity facilitates complex linkages across the food web, including essential symbioses with corals (dotted lines). Major energy transfer pathways from producers to higher levels included consumption by suspension feeders (15 genera), herbivorous reef fish (9 species), and specialist corallivorous fish (3 species). The high richness of invertebrate-eating fish (23 species) further suggests strong top-down control on benthic communities. This metaweb outlines the theoretical blueprint of potential feeding links, framing the subsequent analysis of realized co-occurrence patterns.

### Trophic-dependent Structuring of Biodiversity

Our analyses revealed that biodiversity patterns and their environmental drivers varied systematically across trophic levels, with the influence of environmental filtering diminishing from basal organisms to apex consumers (Figure 3). This trend was evident in both alpha and beta diversity metrics. For instance, the alpha diversity metrics (Figure S5) demonstrated that prokaryotes experienced strong seasonal fluctuations in both species richness and Shannon diversity (Figure S5). In contrast, higher trophic levels exhibited greater stability; benthic fauna maintained consistent richness and diversity year-round, while bony fish diversity (Shannon) remained stable despite seasonal shifts in species richness.

**Figure 3:**
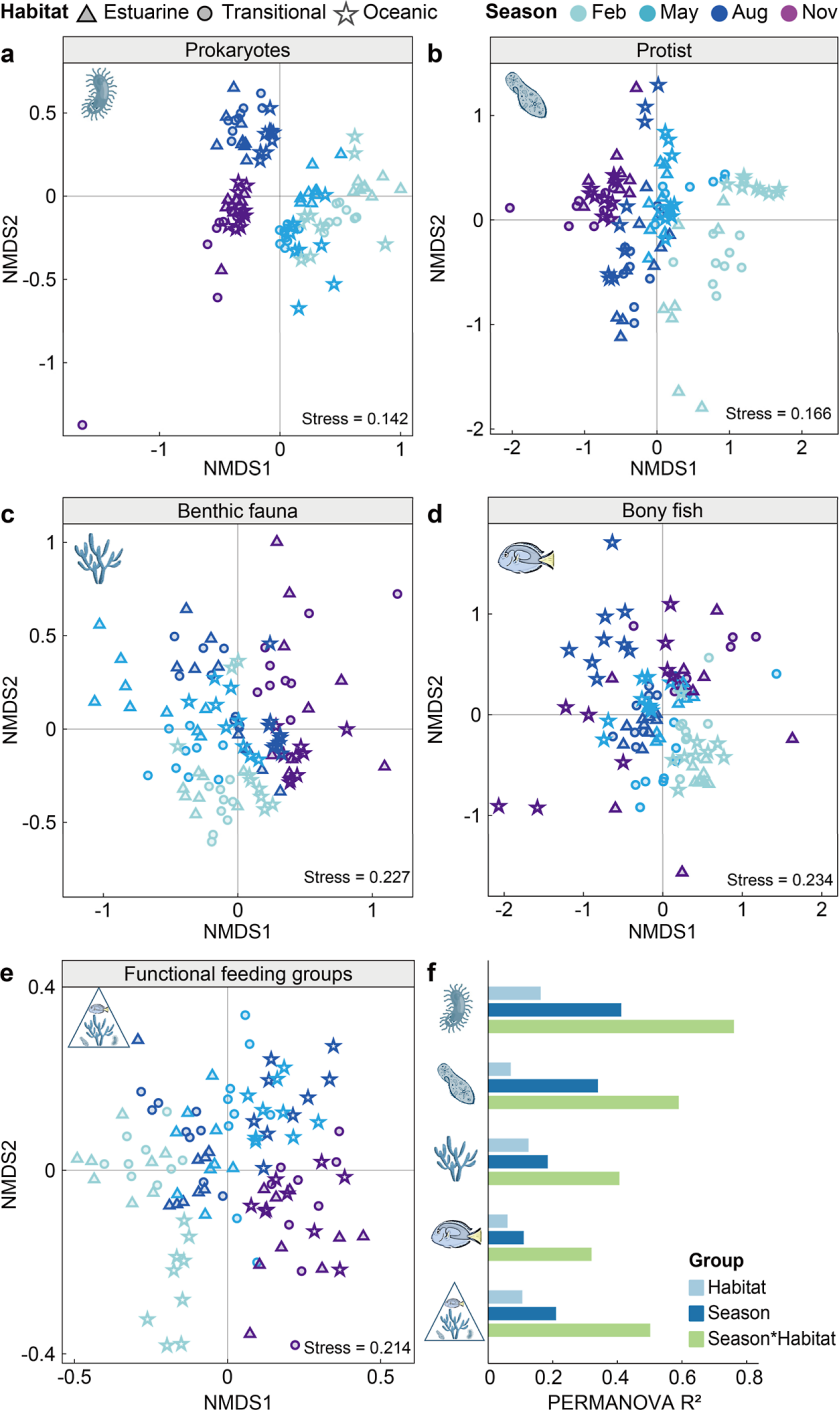
Nonmetric multidimensional scaling (NMDS) analysis showed how community compositions and feeding group structures are shaped by seasons and s. Note: only ASVs >0.01% were used for community compositions. The protist community data was normalized to relative abundance to reduce variance. Permutational multivariate analysis of variance (PERMANOVA) used 9999 permutations and all *p* values less than 0.01.

These differing dynamics were even more pronounced in community composition (beta diversity (Figure 3)). Prokaryotic communities showed clear seasonal clustering (Figure 3a), with separation between cool-season (February and May) and warm-season (August and November) assemblages. A similar seasonal pattern drove protist assemblages (Figure 3b), particularly in oceanic habitat where winter (February) and summer (August) communities were clearly distinct. In contrast, the influence of seasonality diminished for macrofauna. Benthic fauna showed more gradual temporal shifts overlaid with moderate spatial differentiation (Figure 3c). This spatial signal became the dominant structuring force for bony fish, whose assemblages clear separated by habitat (oceanic vs estuarine/transitional), a pattern especially prominent during the summer months (Figure 3d).

Statistical analysis of functional feeding groups quantitatively confirmed this trophic-dependent shift in community compositions (Figure 3e, stress = 0.214). PERMANOVA (Figure 3f) revealed that the interaction between season and habitat explained the most variance for microbial communities (*R* ^2^ > 0.6 for prokaryotes and protists), while spatial effects (habitat) were the primary drivers for benthic fauna (*R* ^2^ > 0.4) and bony fish (*R* ^2^ > 0.3). These trends were consistently supported by ANOSIM results (Figure S6). Crucially, these statistical outcomes demonstrate a systematic weakening of environmental determinism with increasing trophic position: moving from a system dominated by environmental filtering at the base to one increasingly governed by biological traits and mobility at higher levels.

### Environmental Factors Influence Benthic Communities Across Trophic Levels

To elucidate the specific environmental drivers of community composition across trophic levels, we conducted Mantel’s test between community compositions and environmental parameters (Figure 4). Our analyses revealed a clear trophic-dependent gradient in environmental sensitivity. Temperature and salinity emerged as dominant drivers, exhibiting the strongest correlations with prokaryotes and protists (Mantel’s *r* = 0.44, *p* ≤ 0.001). These physical parameters establish the primary environmental template for community development. Dissolved oxygen and turbidity demonstrated moderate correlations with benthic fauna (0.10 < Mantel’s *r* < 0.12) and functional feeding groups (|Mantel’s r| < 0.08), while nutrients (NH_4_, NO_2_, NO_3_) showed weaker yet significant correlations (*p* < 0.05) with most communities.

**Figure 4:**
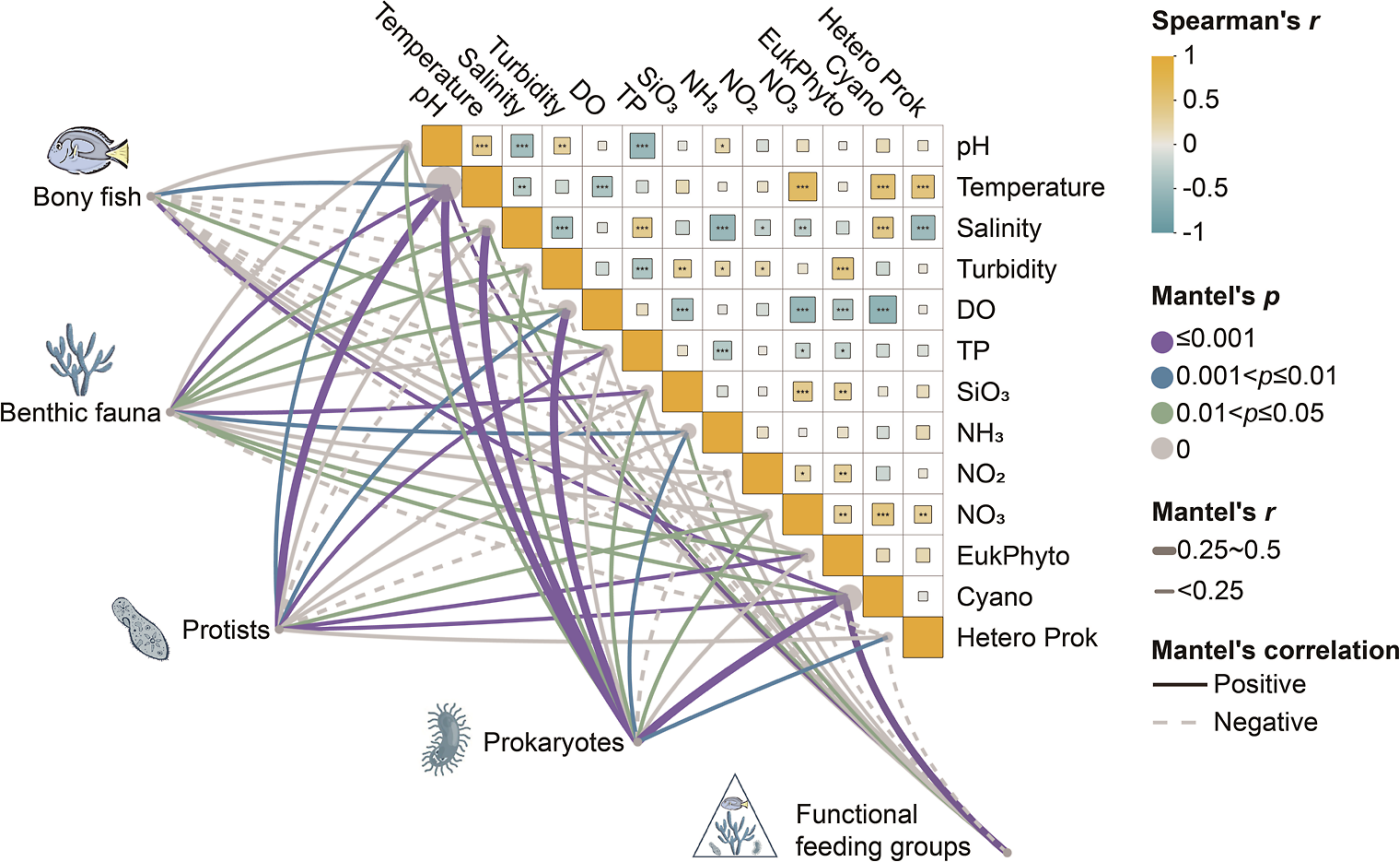
Relationship between environmental factors and compositions of taxonomy (ASV level) and functional feeding groups. Notes: Mantel’s test, Bray–Curtis dissimilarity was used for community composition and Euclidean distance for environmental factors.

Consistent with the NMDS results, the strength of environmental determinism decreased systematically with increasing trophic position. Prokaryotic communities exhibited the highest number of significant correlations with environmental parameters, with strong responses to temperature, salinity, and nutrients. Benthic fauna demonstrated fewer significant environmental correlations, indicating greater independence from direct environmental forcing. Bony fish assemblages exhibited even more selective environmental relationships, with positive correlations with temperature and TP, and negative correlations with turbidity, suggesting their enhanced mobility and active habitat selection capabilities compared to lower trophic organisms.

The analysis further revealed intriguing biotic interactions, with both eukaryotic phytoplankton and cyanobacteria communities showing similar correlation patterns dominated by temperature and nutrient availability. Notably, significant correlations between cyanobacteria and both protist and fish compositions highlighted the importance of indirect trophic interactions. Functional feeding groups displayed intermediate correlation strengths with environmental parameters, integrating multiple direct and indirect responses across constituent taxa. Overall, our analysis demonstrated a clear hierarchical influence of environmental factors, where physical parameters (temperature, salinity) exerted consistently stronger effects than chemical parameters (nutrients) across all taxonomic groups. This progressive attenuation of environmental influence at higher trophic levels supports the hypothesis of a transition toward stronger biotic interactions or stochastic dynamics in structuring upper trophic communities.

### Multitrophic Interactions by Network Analysis

The network analysis of multitrophic co-occurrence patterns revealed distinct spatiotemporal dynamics across the three study habitats (Figure 5). While these networks represent statistical associations rather than confirmed feeding links, they provide comparative metrics of community complexity. The oceanic habitat displayed consistently superior network complexity (derived from six normalized topological parameters), with an exceptional peak in February (0.82 ± 0.07) characterized by densely clustered nodes and numerous strong correlations (Figure 5a, bottom row), particularly among heterotrophic prokaryotes. Though complexity moderated in subsequent seasons, it remained substantially higher than in other habitats. In contrast, the transitional habitat showed similar mild complexity (0.25–0.27) with a distinct August peak (0.38 ± 0.03), while the estuarine habitat exhibited pronounced seasonal variation, from early year lows (0.11–0.14 in February and May) to an August maximum (0.42 ± 0.04).

**Figure 5:**
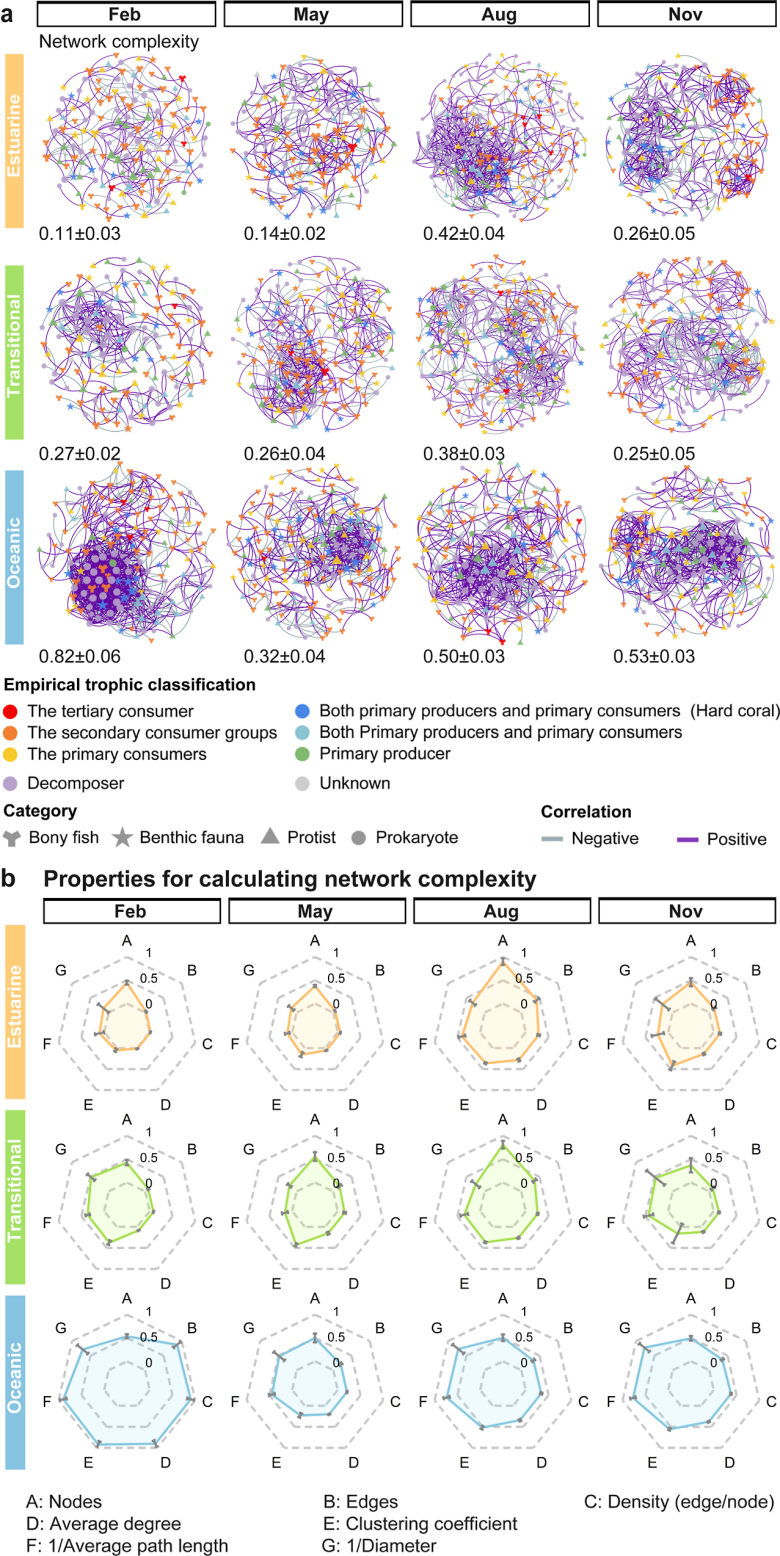
Multitrophic co-occurrence network across seasons and habitats. (a) Visualization of multitrophic co-occurrence networks for three habitats across four sampling periods (February, May, August, November). Network nodes represent taxa from different organismal categories (bony fish, benthic fauna, protist, prokaryote) and are colored according to their empirical trophic classification. Node size is proportional to the number of connections (degree), and edges represent strong correlations (|*r*|≥0.7, *p* < 0.01) between taxa, with purple and gray lines indicating positive and negative correlations, respectively. The network complexity value (mean ± std) displayed below each network represents the mean value of six network properties. (b) Radar plots quantifying six key network properties (A: nodes, B: edges, C: average degree, D: average path length, E: diameter, F: clustering coefficient) that contribute to network complexity calculations. Values are normalized to a 0–1 scale to facilitate comparison across networks. Error bars represent standard deviation.

The radar plots (Figure 5b) revealed systematic spatial differences across all network metrics (nodes, edges, average degree, clustering coefficient, 1/average path length, and 1/diameter). While estuarine and transitional habitats exhibited similar interaction patterns despite environmental differences, the oceanic habitat maintained distinctively higher values across all parameters. This exceptional interconnectedness suggests a structurally stable oceanic ecosystem with established functional redundancy, capable of adaptive reconfiguration in response to temporal environmental shifts. The summer peaks in the transitional and estuarine habitats suggest community reorganization potentially driven by monsoon conditions and increased river discharge.

### Habitat-Specific Environmental Drivers of Multitrophic Network Complexity

Piecewise structural equation models (SEMs) identified distinct, habitat-specific drivers of multitrophic food web complexity across estuarine, transitional, and oceanic habitats. Model fit for each habitat-specific SEM was evaluated using Fisher’s C statistic (Figure 6; all *p*-values >0.05, indicating no significant mismatch between model and data). The selection of predictor variables (temperature, nitrate) was based on variance inflation factors (VIF < 3) to avoid multicollinearity. Thus, we focused on temperature and nitrate as primary environment factors.

**Figure 6:**
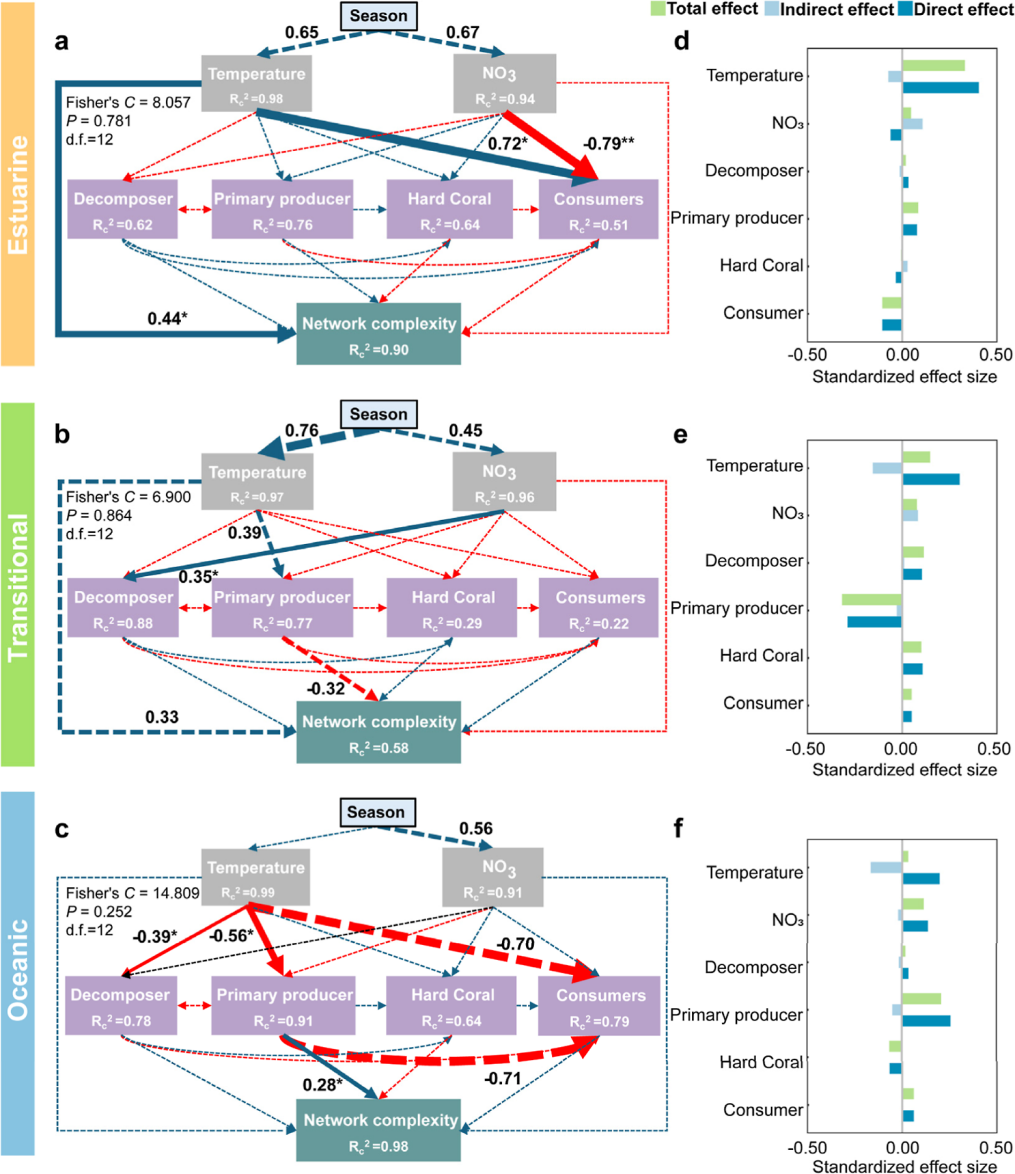
Piecewise structural equation models (SEMs) revealing the drivers of multitrophic network complexity across (a) estuarine, (b) transitional, and (c) oceanic habitats. Path diagrams (a–c) illustrate direct and indirect effects of environmental factors (temperature, NO_3_^–^) and major trophic groups on network complexity. Panels (d–f) show the standardized effect sizes of these relationships. Major trophic groups include decomposers (heterotrophic prokaryotes), primary producers (autotrophic prokaryotes and protists), hard coral, and consumers (cumulative abundance across trophic levels). Blue and red arrows indicate positive and negative relationships, respectively. Solid arrows denote significant relationships (*P* < 0.05), while dashed arrows indicate nonsignificant relationships. Double asterisks (**) indicate *P* < 0.01, and single asterisks (*) indicate *P* < 0.05. Numbers alongside paths represent standardized path coefficients (only coefficients >0.28 are labeled). *R*_c_^2^ (conditional *R* ^2^) represents the proportion of variance explained for each dependent variable in linear mixed model considering random effects. Model formulas are provided in “Code availability.” Box colors denote variable class: light blue stands for “Season”, gray for environmental factors, lilac for major trophic groups, and green for network complexity. Fisher’s C statistics with corresponding *P*-values and degrees of freedom (d.f.) assess overall model fit.

Structural pathways revealed that temperature exerted the strongest direct positive effect on network complexity in estuarine habitats (standardized path coefficient = 0.44), with diminishing influence in transitional (0.33) and oceanic (0.22) zones. This gradient parallels habitat-specific responses in primary producer communities: warming promoted producer abundance in estuarine and transitional habitats, but suppressed it in oceanic waters, reflecting contrasting thermal optima of dominant taxa. Oceanic assemblages were dominated by cold-adapted autotrophic protists (e.g., Chlorophyta), whereas estuarine and transitional zones harbored more warm-adapted cyanobacteria (Figures 1b and S3).

These compositional differences translated into spatially variable effects on network complexity. In oceanic habitats, increased primary producer abundance corresponded with enhanced food web complexity (standardized path coefficient = 0.28), consistent with greater trophic diversity (Figure 6c,f). Conversely, in transitional environments, higher producer abundance associated with reduced network complexity (−0.32), potentially indicating competitive exclusion or decreased niche heterogeneity (Figure 6b,e). Estuarine habitats exhibited minimal direct linkage between producer abundance and network complexity (0.08), suggesting that additional factors mediate complexity in these highly variable systems (Figure 6a,d). The transitional habitat lacked dominant environmental or biotic drivers in the SEM, suggesting a system in flux governed by complex variables beyond standard regulation. Although environmental constraints on consumer abundance varied spatially, consumers consistently exerted negligible direct influence on network complexity across all habitats (|path coefficients| < 0.12). Collectively, these results suggest environmental drivers and primary producer dynamics play more prominent roles in shaping multitrophic network complexity than consumer-mediated processes.

## Discussion

By integrating eDNA data with multitrophic network analysis and structural equation modeling (SEM), our study disentangles the complex forces governing community assembly in Hong Kong’s urbanized coastal ecosystem. We reveal a dynamic interplay between environmental filtering and biotic regulation, where the strength of environment determinism systematically wanes at higher trophic levels. Crucially, our results demonstrate that the mechanisms maintaining ecosystem stability are not universal but are habitat-specific, representing fundamentally different modes of ecosystem organization. These findings provide a novel, multitrophic perspective on how biodiversity is structured in complex, human-impacted marine environments.

### A Trophic Gradient in Environmental Determinism

A key finding of this study is the systematic decline in environmental control with ascending trophic level (Figure 3), statistically evidenced by the decreasing variance explained by environmental factors (PERMANOVA *R* ^2^). At the base of the food web, prokaryotic (*R* ^2^ = 0.76) and protist (*R* ^2^ = 0.59) communities were tightly coupled to physical parameters, particularly temperature and salinity (Figure 4). This strong environmental filtering aligns with metabolic theory, which posits that smaller organisms with faster generation times and higher surface-area-to-volume ratios are more directly and rapidly influenced by ambient environmental conditions.^20^ Similar patterns have been observed in diverse aquatic systems, from temperate lakes to river networks,^38,39^ suggesting that the strong bottom-up control by the physical environment on microbial communities is a general ecological principle. In our highly dynamic subtropical system, this manifests as pronounced seasonal turnover, positioning these microbial assemblages as sensitive bioindicators of environmental change.

In contrast, higher trophic organisms such as benthic fauna (*R* ^2^ = 0.41) and fish (*R* ^2^ = 0.32) displayed a marked decoupling from these direct physical drivers. Instead, their community compositions were more selectively influenced by habitat features (e.g., turbidity) and resource availability. (Figure 4). This decoupling reflects how mobility and behavioral habitat selection allow higher consumers to buffer direct physical fluctuations. Importantly, the significant correlations between microbial groups and fish composition (Figure 4) reveal how environmental influence propagates upward: environmental effects, directly imprinted on the microbial base, are attenuated and translated into resource-driven patterns that shape higher trophic levels. This supports the “asymmetric propagation of disturbance” concept in meta-ecosystem theory,^40^ where the spatial subsidies of energy and biomass connect disparate community modules. Although a fish can leverage its mobility to mitigate acute environmental stressors, its persistence is ultimately governed by the productivity of the food web it depends on. Therefore, the trophic gradient marks a transition from direct environmental filtering at the base to the dominance of biotic interactions and habitat choice at the top.^41,42,88^

### Habitat-Specific Mechanisms of Ecosystem Stability

This trophic gradient in environmental control translates into fundamentally different mechanisms of ecosystem organization across habitats, as revealed by contrasting network structure and stability patterns that are further supported by our SEM analysis (Fisher’s C, *P* > 0.05). The highest levels of network complexity and robustness were observed in the oceanic habitat (Figure 5). Here, SEM results indicate that network complexity is primarily governed by internal biotic dynamics (Path Coefficient >0.28), specifically the abundance and diversity of primary producers. This pattern highlights how biodiversity and ecosystem engineering jointly sustain resilience,^43,49^ representing a self-regulating system where biological feedbacks buffer environmental variability. This biotic regulation is exemplified by hard corals (e.g., *Favites*, *Goniopora*), which provide critical structural complexity^44^ and foster high functional redundancy among groups like heterotrophic prokaryotes and omnivorous fish (Table S7A,B). While classic ecological theory suggests that high connectance can amplify disturbance propagation,^45,46^ the stability of the oceanic network aligns with recent findings that nonrandom structures provided by ecosystem engineers can buffer ecosystems against perturbations.^47^

Conversely, the estuarine habitat, subject to strong riverine influence, exhibited a simpler, more fragile network. Here, network dynamics were dictated by the dominance of external abiotic forcing. Our SEM results identified temperature as the primary controller of network complexity, likely because it stimulates basal metabolic and microbial processes.^56^ This strong abiotic control, combined with lower functional redundancy, creates a system with diminished resilience that is highly vulnerable to external perturbations.^48^ The transitional habitat represented an intermediate state, where the absence of a single dominant driver in our SEM suggests that its dynamics may be governed by a complex interplay of competing factors or a higher degree of unmeasured stochastic processes.^49,50^ Collectively, these findings indicate that the mechanisms governing ecosystem stability are context-specific. In stable environments, biotic interactions confer resilience, while in fluctuating habitats, system fragility is a direct consequence of its control by abiotic forcing.^51^

### Functional Redundancy as a Mechanism for Resilience

This context-dependent stability is fundamentally underpinned by functional redundancy, which provides the mechanistic basis for resilience across the environmental gradient. Our trophic metaweb (Figure 2) revealed several keystone functional groups that confer this resilience. For example, the seasonal succession of primary producers, ranging from winter-dominant *Ostreococcus* to spring-prevalent *Synechococcus* CC9902, ensures continuous productivity by providing alternative pathways for energy flow as environmental conditions change. This functional compensation among taxa with different ecological niches is a hallmark of resilient systems,^52^ and similar multitrophic network structures have been shown to buffer other marine systems against disturbances.^53,54^ Similarly, the high prevalence of mixotrophic protists provides a crucial stabilizing feedback by maintaining energy transfer during periods of resource fluctuation.^55,56^ Additionally, hard corals contribute to network robustness through their dual capacity as both structural ecosystem engineers^57,58^ and trophically plastic consumers.^59^ The spatial partitioning of different coral genera, such as stress-tolerant *Oulastrea* in transitional waters and light-dependent *Pavona* in oceanic habitats, illustrates niche specialization that maximizes ecosystem function across the environmental gradient. However, the scarcity of these key habitat-formers in the turbid western waters serves as a critical warning that this buffering capacity has finite limits and can be eroded by cumulative stress.^60,61,88^ This spatial partitioning extends to reef fish, with herbivorous *S. fuscescens* dominating turbid habitats while planktivorous *S. gracilis* dominates clear oceanic sites, collectively sustaining ecosystem function across the gradient.

## Limitations and Conclusions

While this study advances our understanding of coastal ecosystem stability, several methodological constraints should be acknowledged. First, although the reconstruction of ecological networks from eDNA metabarcoding data is a powerful tool for revealing community patterns; we recognize that relative sequence abundance is not a direct proxy for biomass or interaction strength. Therefore, these inferred networks must be corroborated by more direct ecological measurements. Second, the co-occurrence networks derived here reflect statistical associations and do not definitively confirm the direct trophic interactions mapped in the conceptual metaweb. We interpret these approaches complementarily: the metaweb outlines the theoretical blueprint of potential feeding links, while the co-occurrence networks reveal how these associations are realized under environmental filtering. Furthermore, while the network metrics used here (e.g., modularity, path length) are ubiquitous in microbial ecology, they are equally foundational in macroscopic food web theory for assessing community stability and compartmentalization. Future research should ideally integrate controlled experiments or higher-resolution temporal series to causally validate the mechanistic relationships proposed here.

Despite these limitations, our findings provide a robust scientific basis for developing targeted conservation strategies. Our research demonstrates that the resilience of urban coastal ecosystems arises from a dynamic hierarchy of controls that shifts across environmental gradients. At a broad scale, environmental filtering strongly shapes foundational microbial communities. This influence attenuates at higher trophic levels, where biotic interactions become more prominent. Crucially, the dominant regulatory mechanism shifts depending on local conditions, from the dominance of external abiotic forcing in volatile systems to governance by internal biotic dynamics in stable ones. These insights highlight the importance of management approaches that account for this shifting hierarchy of controls, particularly the protection of network hubs and functional redundancy, which together offers a practical framework for safeguarding coastal ecosystems under increasing anthropogenic pressure.

## Methods

### Sample Collection

To ensure comprehensive data collection, we implemented a quarterly sampling approach for both eDNA water samples and visual surveys of coral and fish communities over a continuous annual cycle in 2023 (Feb, May, Aug and Nov). This sampling was conducted across nine strategically selected sites spanning the study habitat’s environmental gradient (Figure 1 and Table S1). At each site, we established three replicate 30 m-long benthic transects over coral habitats. All samplings were conducted at benthic depth (3.3 ± 1.8 m) to ensure direct comparability between the eDNA and underwater visual census (UVC) data sets. From the midpoint of each transect, we collected a 5 L integrated water sample using acid-cleaned LDPE bags. The transect deployment strategy followed a systematic approach: two initials transect were positioned in opposite directions from a starting point marked with a piece of rebar, with the third transect placed perpendicular to create comprehensive spatial coverage of the coral community. The three transects were treated as replicates for each site. In areas where coral communities were restricted to shallow depths (<3 m) with narrow vertical distribution, we modified this approach by placing the third transect as an extension from one existing transect, ensuring a minimum 10 m separation between transect end points to prevent redundant sampling of the same coral community areas.

For eDNA analysis, a 1 L aliquot from each sample was immediately filtered through a 0.45 μm Sterivex-HV pressure filter unit (Cat. no. SVHV010RS, Millipore, U.S.A.), resulting in three filtered replicates per site per season. Thus, a total of 3 L of seawater was filtered per site per season. Across the entire study (9 sites × 4 seasons × 3 replicates), this yielded 108 eDNA samples. To monitor potential contamination, field negative controls were processed following identical procedures at each sampling event. All Sterivex filter units were flash-frozen on dry ice to preserve eDNA integrity and subsequently transferred to −80 °C storage until DNA extraction. For complementary analyses, we collected additional water sample fractions: unfiltered water was preserved in acid-cleaned brown bottles on ice for cell counting procedures, while 0.45 μm filtered water was retained for macronutrient measurement.

### Underwater Visual Surveys

We conducted simultaneous underwater visual surveys along the same transects to document fish and coral communities during water sample collection.^62,65^

Underwater visual surveys for fish were conducted as described before^63^ by a single trained observer to eliminate interobserver variability. This traditional visual survey is a widely adopted and reliable method used worldwide.^64–66^ The observer navigated each transect at a consistent height (∼1 m above substrate) and speed (∼5 m/min), methodically documenting all fish encountered within 2 m on either side of the transect line when water visibility permitted. Fish total body length was estimated visually to the nearest cm, except for specimens <1 cm which were measured to the nearest mm (0.1 cm). This standardized approach yielded a total surveyed area of 360 m^2^ per site per season (three 30 m × 4 m belt transects). Throughout all surveys, the observer was equipped with an Olympus Tough TG-6 digital camera in underwater housing to photograph unidentified or taxonomically challenging specimens for postsurvey verification. Taxonomic identification was performed to species level whenever possible, with genus or family level classification applied only when species-level identification was precluded by poor visibility or ambiguous morphological characteristics.

Immediately after the completion of fish survey for a transect, benthic video footage was taken using a framer that mounts a GoPro Hero camera in underwater housing, set perpendicular to and at a constant height of ∼1 m above the benthos. Postsurvey, photoquadrats were manually segmented at 1 m interval from the video footages for each transect, resulting in a total of 3180 photoquadrats (30 images × 3 transects × 9 sites × 4 seasons, minus 2 transect surveys that were not performed for Peng Chau in August 2023 due to poor weather condition; Table S4). Each photoquadrat was then manually annotated and analyzed for benthic community composition, in the form of percent cover, on ReefCloud (https://reefcloud.ai; accessed in April-October 2024). A total of 50 points were randomly overlaid to each photoquadrat while annotating, where 30 points were manually annotated by a single researcher and the other 20 points were annotated by the ReefCloud’s machine learning model. Due to low consistency between human and computer annotations, only 30 human annotations were used for characterization of benthic community composition. Major biotic and abiotic benthic groups (e.g., black coral, gorgonian, sponge, macroalgae, sand, rock, rubble) were identified. A finer identification to genus level with notes on their health status (i.e., live and dead) were done specifically for hard corals. Finally, man-made items and unidentifiable or unknown objects were separately labeled as “Others-Abiotic” and “Unknown/Unidentifiable” in the analysis.

### Metadata Collection

Sampling depth, water temperature, salinity, chlorophyll, turbidity, pH, and dissolved oxygen were measured in situ using multiparameter sondes (YSI EXO3, Yellow Springs Instruments, USA; ASTD-102 RINKO-Profiler, Japan). Levels of nutrients, including ammonia nitrogen (NH_4_^+^), nitrate (NO_3_^–^), nitrite (NO_2_^–^), silicate (SiO_3_^2–^), total phosphorus (TP), and orthophosphate phosphorus (PO_4_^3–^) were analyzed using spectroscopy (colorimetry and photometry) methods following the APA standard procedures.^67^ Water subsamples were fixed by glutaraldehyde (final concentration: 0.25%) and analyzed using flow cytometry (FCM) (BD FACSCelesta, BD Biosciences, USA) to enumerate the absolute abundance of total phytoplankton, phototrophic eukaryotes (Euk Phyto), cyanobacteria (Cyano), and heterotrophic prokaryotes (Hetero Prok) following the established protocols.^68^ The detailed results are shown in Figure S9.

### eDNA Extraction

The eDNA captured on Sterivex-HV pressure filter units was extracted using the Qiagen DNeasy Tissue and Blood DNA extraction kit (Qiagen, German) following a modified protocol optimized for aquatic environmental samples.^69^ To monitor contamination throughout the workflow, procedural control samples consisting of Milli-Q water were processed in parallel with field samples through all stages: sample collection, filtration, DNA extraction, and PCR amplification. The extraction protocol employed a buffer-based cell lysis approach. For each filter unit, we prepared a lysis solution containing 220 μL PBS, 20 μL proteinase K, and 200 μL buffer AL. This solution was gently pipetted into the Sterivex cartridge. Loaded cartridges were then incubated at 56 °C for 60 min with continuous shaking at 120 rpm. Following incubation, the lysate was carefully collected from each cartridge and processed according to the manufacturer’s spin column purification protocol. The final elution was performed with 60 μL of elution buffer. DNA yield and purity were assessed using NanoDrop spectrophotometers and Qubit 4 fluorometer (Thermo Fisher Scientific Inc., USA).

### eDNA Metabarcoding

We implemented a multimarker metabarcoding approach following the early pooling protocol.^70^ Five distinct primer sets targeting different taxonomic groups were employed to comprehensively profile marine biodiversity from the collected environmental DNA samples. We used the Platinum SuperFi II PCR Master Mix, as its formulation allows for a single, unified annealing temperature across all barcoded primer sets. Each 20 μL first-round PCR reaction contained 10 μL Platinum SuperFi II PCR Master Mix, 2 μL barcoded forward primer (5 μM), 2 μL barcoded reverse primer (5 μM), 0.8 μL BSA (10 mg/mL), 3.2 μL ultrapure molecular grade water, and 2 μL DNA template. The primer sets included MiFish-U for bony fish,^71^ two ITS2 primer sets for corals (CoralITS2 for most scleractinians excluding Acropora^72^ and CoralITS2_acro specifically for Acropora^14^), eukaryotic 18SV4 SSU primer set for protists,^73^ and 515Y-926R 16SV4 V5 for prokaryotes.^74,75^ The detailed PCR conditions and primer sequences are listed in Table 1.

**Table 1:** Metabarcoding Primers and PCR Conditions^a^

primer assay	targets	primer sequence (5′ to 3′)	PCR condition
MiFish-U	bony fish	F: GTCGGTAAAACTCGTGCCAGCR: CATAGTGGGGTATCTAATCCCAGTTTG	initial denaturation at 98 °C for 30 s, followed by 35 cycles of 10 s at 98 °C, 10 s at 60 °C, and 15 s at 72 °C, and a final extension for 4 min at 72 °C
coral ITS2	benthic fauna (no Acropora)	F: GARTCTTTGAACGCAAATGGCR: GCTTATTAATATGCTTAAATTCAGCG	
coral ITS2_acro	benthic fauna (with Acropora)	F: GARTCTTTGAACGCAAATGGCR: TCGCCGTTACTGAGGGAATC	
eukaryotic 18SV4 SSU	protists	TAReuk454FWD1: CCAGCASCYGCGGTAATTCCTAReukREV3: ACTTTCGTTCTTGATYRA	
515Y-926R 16SV4 V5	prokaryotes (bacteria and archaea)	515F-Y: GTGYCAGCMGCCGCGGTAA926R: CCGYCAATTYMTTTRAGTTT	

a A unified annealing temperature of 60 °C was used for all primer sets.

Following previous study,^70^ libraries were constructed via a two-step PCR approach. The first round of PCR products (“tagged” PCR products) was visualized using gel electrophoresis to confirm successful amplification and absence of contamination in negative controls. First round of PCR products (“tagged” PCR products) was pooled in equimolar ratios and purified using exonuclease ExoSAP-IT PCR Product Cleanup Reagent (Applied Biosystems) prior to the second-round PCR,^70^ which incorporated sequencing adapters and indices. All negative controls showing no amplification were excluded from the pooling step. A second index PCR was then performed to append sequencing adapters, followed by purification with AMPure XP beads (Beckman Coulter) and size selection using the E-Gel SizeSelect systems (Invitrogen). Final libraries were sequenced by Novogene (Beijing, China) using Illumina Novaseq XPlus with 2 × 150 bp paired-end chemistry for MiFish-U libraries and 2 × 250 bp paired-end chemistry for the longer amplicons from other markers. Data quality remained robust, with Q30 scores exceeding 90% across all samples.

## Bioinformatics

The detailed bioinformatic pipeline is summarized in Supporting Information Table S3 and available as executable code at https://github.com/shanexuuu/YUNGlab-eDNA2023 (in data availability section). Key steps included: quality filtering and primer trimming using cutadapt; denoising with DADA2 to obtain amplicon sequence variants (ASVs); taxonomic assignment against curated reference databases (SILVA 138 for prokaryotes, PR2 for protists, custom databases for fish and corals); removal of potential contaminants, and postclustering curation etc. Postclustering curation and SSU rRNA read correction details are provided in Tables S8–S13 and Figures S7 and S8. Metaweb construction. We constructed comprehensive marine trophic metaweb based on taxonomic assignments derived from multimarker eDNA metabarcoding data and existing knowledge of interactions. Taxonomic resolution was maintained at the species level for fish (using MiFish-U markers) and at the genus level for other taxonomic groups to balance precision with ecological interpretability. Trophic interactions were modeled following established feeding relationships among marine functional groups as described in recent literature.^76,77^

To address trophic complexity, we incorporated current understanding of functional feeding ecology Scleractinian corals were classified as both primary producers and primary consumers to reflect their well-documented trophic plasticity across environmental gradients.^78–80^ This dual classification acknowledges their nutritional derivation from both photosynthetic endosymbionts and heterotrophic feeding.

For network construction, amplicon sequence variants were consolidated at the genus level for most taxa, while fish molecular operational taxonomic units were aggregated at the species level. The resulting food web incorporated 407 distinct taxa assigned to 20 functional feeding groups (including two with undetermined feeding strategies). Complete trophic classifications and network structures are detailed in Tables S2 and S5.

### Multitrophic Co-occurrence Network

To investigate ecological associations, we constructed co-occurrence networks based on taxon correlations across 12 distinct season-habitat combinations. To balance taxonomic precision with ecological relevance, analyses were conducted at the genus level for most organisms, while species-level resolution was preserved for fish.^81^ Correlations were calculated using FastSpar v1.0.0, which implements the SparCC algorithm designed for compositional data.^82,83^ This approach enables robust detection of significant associations across trophic levels under seasonal and spatial influences. While these networks do not confirm direct biological interactions, they can reveal the realized trophic and nontrophic associations that structure a community. This approach therefore allows us to map how the community is shaped in practice by the interplay of environmental gradients and biological processes across seasons and habitats. Using the R package igraph, we constructed 12 networks and individual sample subnetworks through the “graph.adjacency” and “subgraph” functions.^84^ Network topology was characterized through multiple metrics: node and edge counts, density (node counts/edge counts), average degree, clustering coefficient, average path length, and network diameter. Higher values of nodes, edges, density, average degree, and clustering coefficient, coupled with lower values of path length and diameter, indicate stronger network connectivity and increased network complexity.^85,86^ While these metrics are ubiquitous in microbial ecology, they are equally foundational in macroscopic food web theory for assessing community stability and compartmentalization.^6,87,88^ We applied min–max normalization to each parameter, then calculated their mean value to create a composite index representing overall network complexity.^86^ Networks were visualized using the online platform ChiPlot (available on https://www.chiplot.online/) and radar plot was visualized employing Origin software, version 2018. The topological significance of each node was evaluated using its within-module connectivity (Zi) and intermodule connectivity (Pi) metrics from R library “brainGraph” v3.1.0. Specifically, Zi quantifies how well a node is connected to other nodes within the same module, whereas Pi measures its connections to nodes in different modules.^89^ Based on these Zi and Pi values, each node’s topological role was categorized into four distinct groups: 1) network hubs (Zi > 2.5 and Pi > 0.62); 2) module hubs (Zi > 2.5 and Pi ≤ 0.62); 3) connectors (Zi ≤ 2.5 and Pi > 0.62); and 4) peripherals (Zi ≤ 2.5 and Pi ≤ 0.62). Nodes identified as network hubs, module hubs, or connectors were considered topologically important (TI) taxa.^86^

### Structural Equation Model

We implemented structural equation models (SEM) using the piecewiseSEM package v2.3.0.1 ^90^ in R v4.4.2. All variables were normalized using the bestNormalize function from the bestNormalize package^91^ to satisfy model assumptions of normality and residual homogeneity. Each causal pathway was tested using linear mixed models that incorporated random intercept effects for season and site. Model fit was evaluated using Fisher’s C statistic.^92^ Indirect effects were quantified by multiplying standardized coefficients along the model pathways.

### Statistical Analysis

We used the R vegan package v 2.6.10 to calculate the alpha diversity and perform the analysis of similarities (ANOSIM) with 9999 permutations, defining groups by Site, Season, Habitat, and Season × Habitat. For the ANOSIM, significant group differentiation was defined by an *R*-value >0 and a *p*-value <0.05 ^76^. We then performed the indicator species analysis using the *R* indicspecies package v 1.8.0,^93^ with *p*-value cutoff lower than 0.05 and indicator value (IndVal) higher than 0.50. As a well-established method for identifying characteristic taxa in ecological studies,^94–97^ the results of such analysis must be contextualized within the specific sampling design and threshold reliance. We therefore used this analysis to infer the habitat-season preference of taxa in each multitrophic network. For data visualization and analysis including Mantel’s test, heatmap, nonmetric multidimensional scaling (NMDS), permutational multivariate analysis of variance (PERMANOVA) with 9999 permutations across groups defined by Season, Habitat, and Season × Habitat were generated and visualized using the online platform ChiPlot (available on https://www.chiplot.online/).^98^

## Supplementary Material





## Data Availability

All raw sequence data generated in this study have been deposited in both the Genome Sequence Archive (Chen et al., 2021) of the National Genomics Data Center (CNCB-NGDC Members and Partners, 2022) at the China National Center for Bioinformation/Beijing Institute of Genomics, Chinese Academy of Sciences, and the National Center for Biotechnology Information (NCBI). These data are publicly accessible at https://ngdc.cncb.ac.cn/gsa under accession number PRJCA039211 and at NCBI under the BioProject accession number PRJNA1257527. All bioinformatic processing scripts (Linux bash) and statistical analysis code (R) are available in our public GitHub repository at https://github.com/shanexuuu/YUNGlab-eDNA2023.

## Notes

Comparison of traditional visual survey data and flow cytometry data with eDNA metabarcoding results; Diversity of community compositions; Comparison of rRNA correction; Environmental data; Rarefaction curves (PDF)
And data sets used in this study (XLSX) (XLSX)
