# Supplementary material for: Environmental Filtering Weakens with Trophic Level in Urban Coastal Ecosystems
Source: Environ Sci Technol. 2026 Jan 30;60(5):4042–57. doi: 10.1021/acs.est.5c08142 (PMC12915753; doi:10.1021/acs.est.5c08142)
Supplement: Supplementary file 1 [file es5c08142_si_001.pdf]

Supplementary information

Environmental filtering weakens with trophic level in urban coastal ecosystems

Wenqian Xu[1], Yu-De Pei[1], Taylor M.W. Li[1], Joshua Bennett-Williams[1], Ruixian Sun[1], Shara K.K. Leung[1], Masayuki Ushio[1], Alex S. J. Wyatt[1*], Charmaine C.M. Yung[1*]

Affiliations:
[1] *Department of Ocean Science, The Hong Kong University of Science and Technology, Hong Kong SAR. China*

***Corresponding authors:**
Charmaine C.M. Yung (ccmyung@ust.hk)
Alex S. J. Wyatt (wyatt@ust.hk)

This 11-page document includes 9 supporting figures (Figure. S1- S9):

Figure S1. Rarefaction curves of all samples.

Figure S2. Benthic fauna comparison, with (a) visual survey data on benthic coverage and (b) eDNA ITS results at genus level.

Figure S3. Bony fish assemblages' comparison at species level, with (a) visual survey data and (b) eDNA MiFish-U results.

Figure S4. Linear regression analysis comparing eDNA sequence reads with flow cytometry (FCM) data for (a) cyanobacteria, (b) heterotrophic prokaryotes, and (c) eukaryotic phytoplankton.

Figure S5. Alpha diversity of rarefied reads at ASV or MOTU level.

Figure S6. ANOSIM results of community compositions at ASV level. Note: all p values were lower than 0.01, which means the group separation was significantly meaningful.

Figure S7. Comparisons of alpha and beta diversities after corrections. Note: R2 and P value mean the PERMANOVA results group by seasons in (b).

Figure S8. Comparison of phylum compositions between correction of 16SV4V5 prokaryotic reads and 18SV4 protistan reads

Figure S9. Environmental factors and cell abundances across regions and seasons.

Supplementary Figures

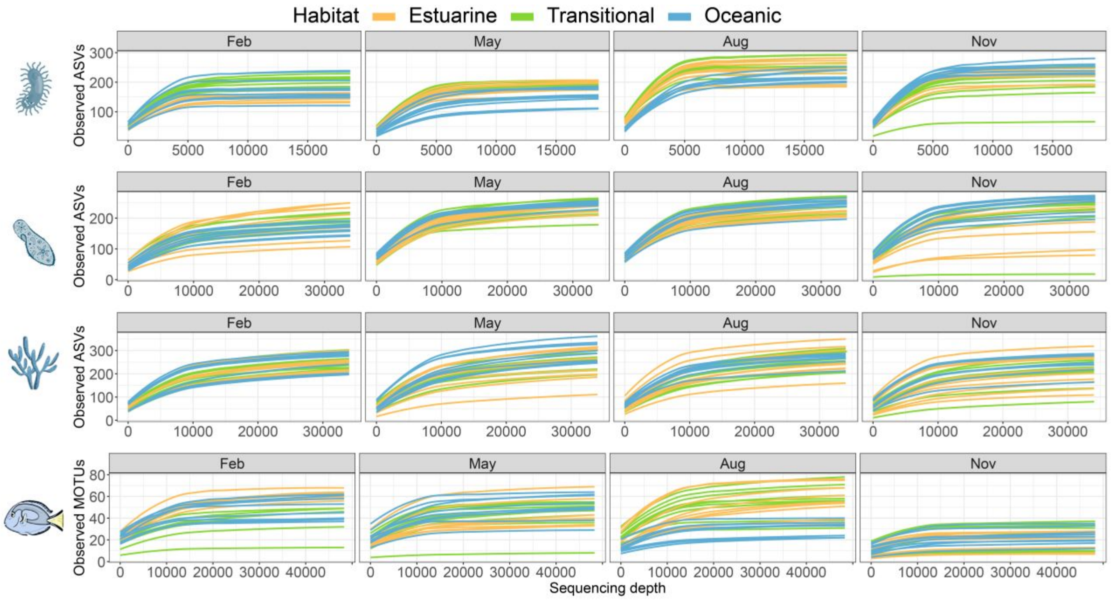

Figure. S 1 Rarefaction curves of all samples.

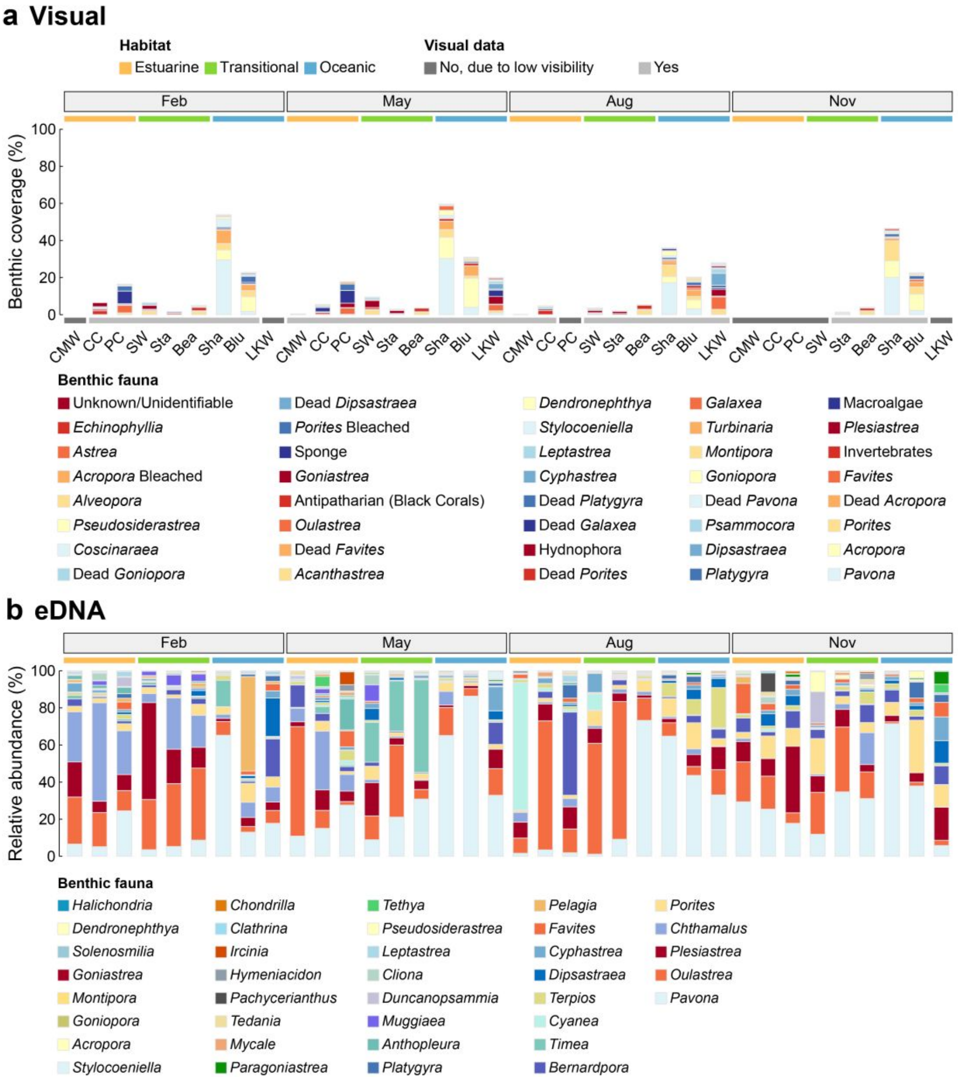

Figure S 2. Benthic fauna comparison, with (a) visual survey data on benthic coverage and (b) eDNA ITS results at genus level. Note: In (a), only biotic components are depicted; and the rest components were abiotic like sand and rock. The ranking of benthic fauna from bottom to top reflects their mean values from highest to lowest across all samples.

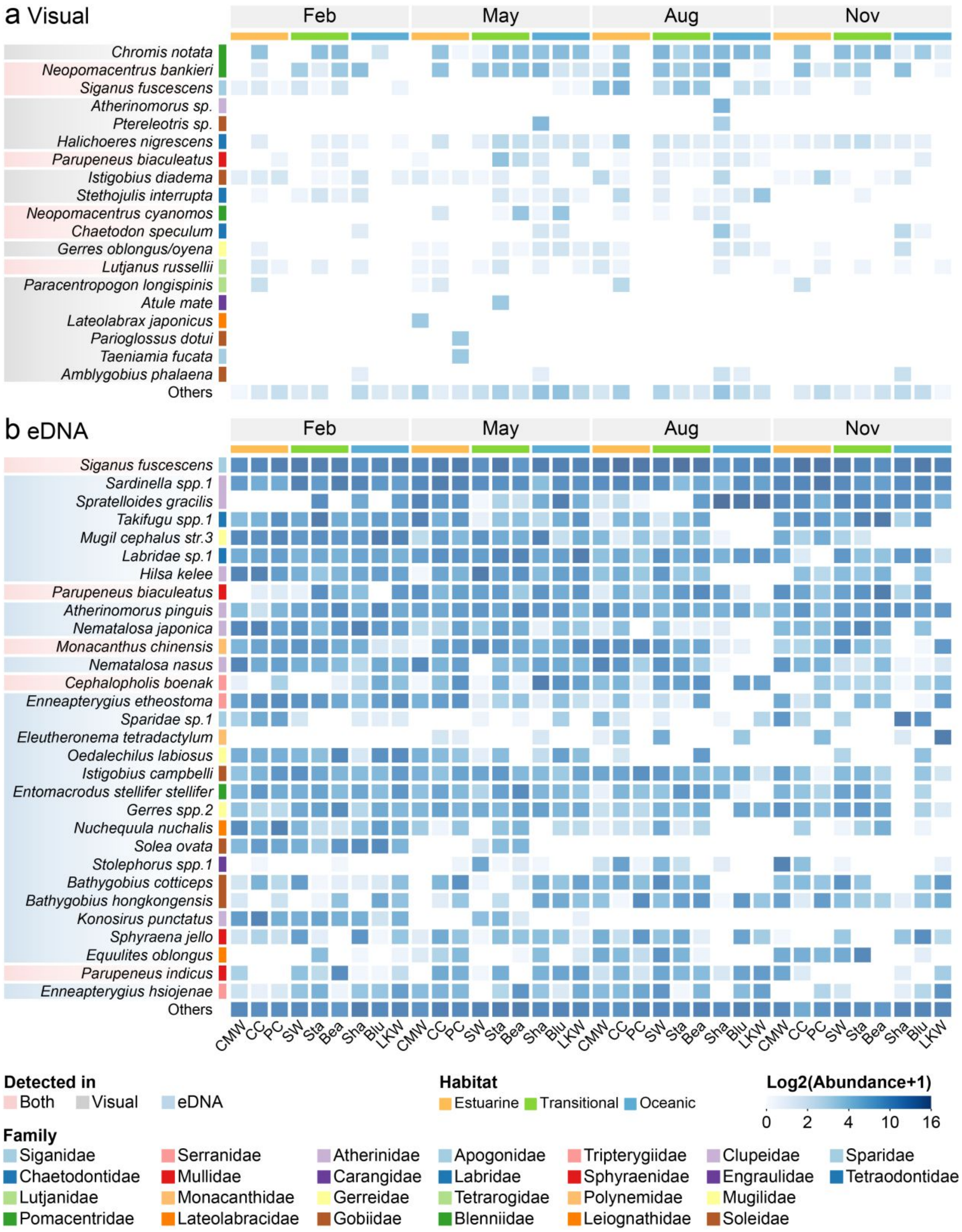

Figure S 3. Bony fish assemblages' comparison at species level, with (a) visual survey data and (b) eDNA MiFish-U results. Note: In (a), no visual data for PC_Aug due to ultra-low visibility. The ranking of bony fish species from top to bottom reflects their mean relative abundance from highest to lowest across all samples. Only the mean relative abundance higher than 0.5% were shown.

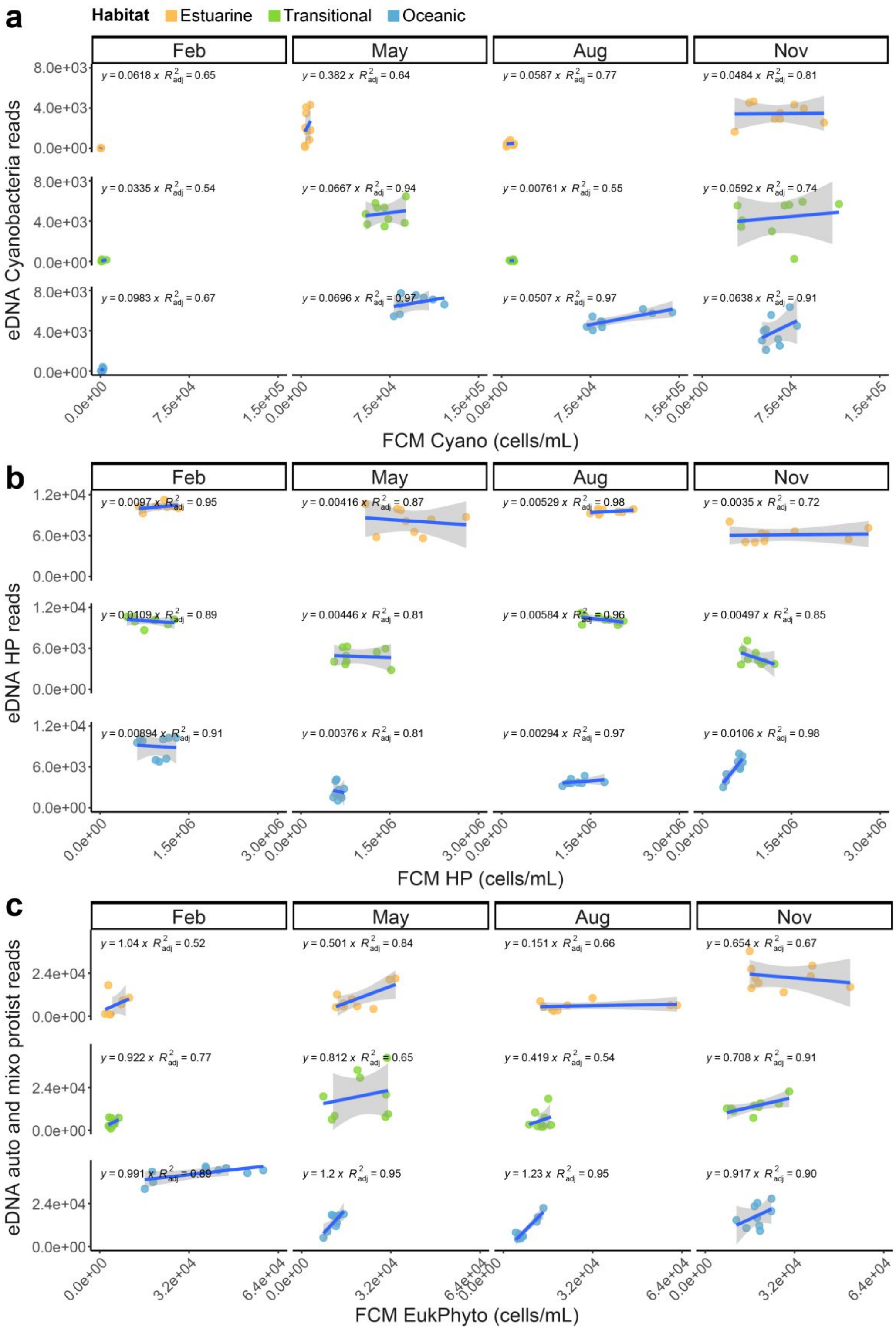

Figure S 4. Linear regression analysis comparing eDNA sequence reads with flow cytometry (FCM) data for (a) cyanobacteria, (b) heterotrophic prokaryotes, and (c) eukaryotic phytoplankton. In FCM data, "Cyano" indicates cyanobacteria abundance, "HP" denotes heterotrophic prokaryotes, and "EukPhyto" represents eukaryotic phytoplankton. For eDNA, "auto and mixo protist" refers to autotrophic and mixotrophic protists, which are assumed to possess chloroplasts and thus are measurable by FCM. The regression models assume zero interception, reflecting the notion that sequence reads are zero in the absence of specific cells.

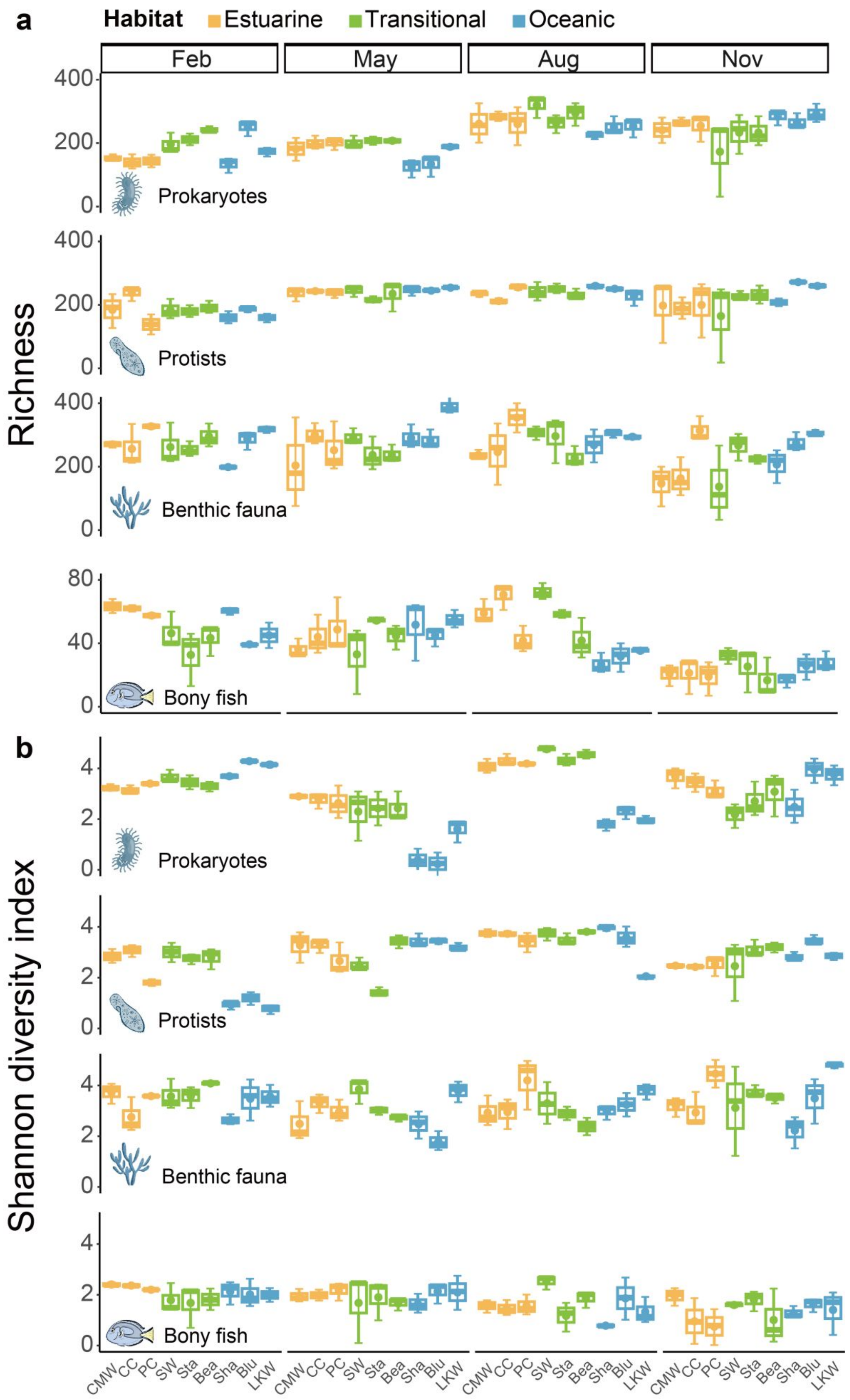

Figure S 5. Alpha diversity of rarefied reads at ASV or MOTU level.

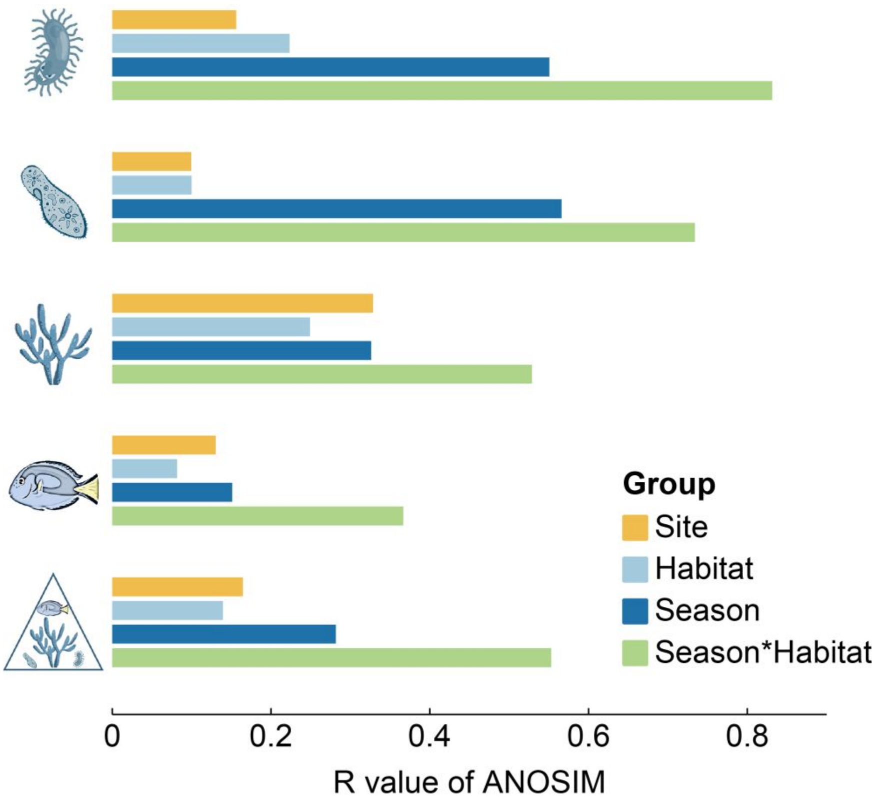

Figure S 6. ANOSIM results of community compositions at ASV level. Note: all p values were lower than 0.01, which means the group separation was significantly meaningful.

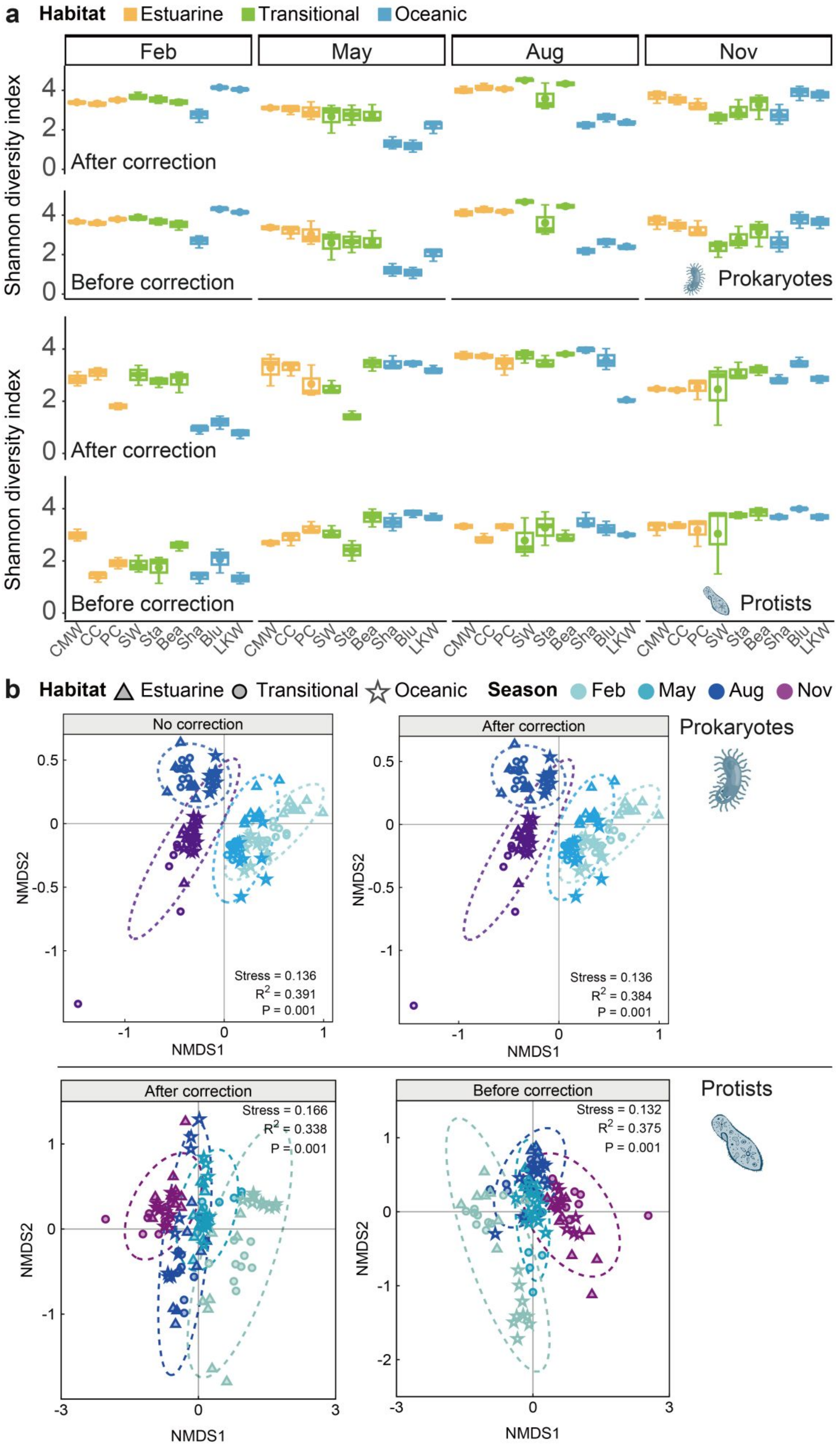

Figure S 7. Comparisons of alpha and beta diversities after corrections. Note: R^2 and *P* value mean the PERMANOVA results group by seasons in (b).

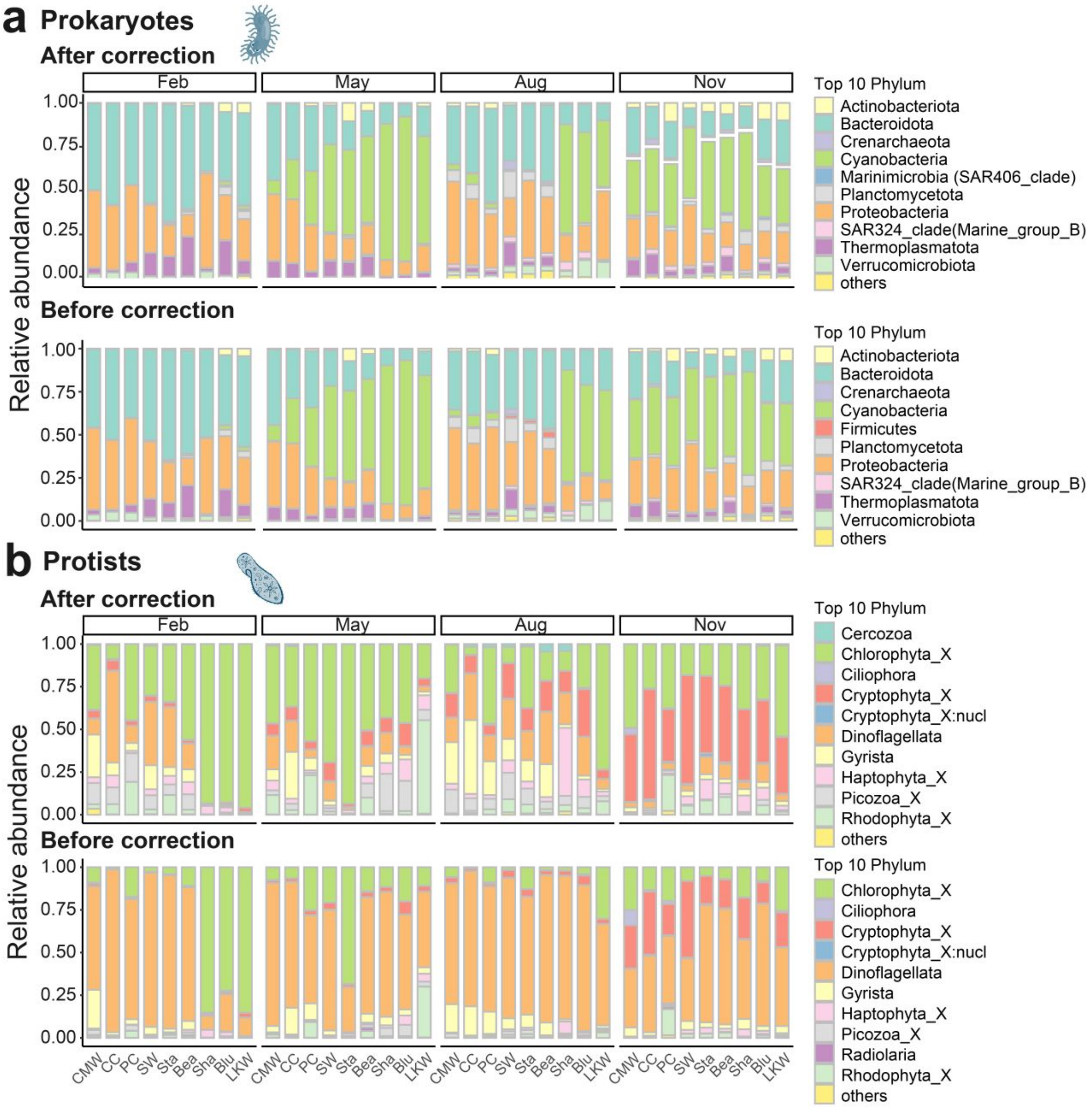

Figure S 8. Comparison of phylum compositions between correction of 16SV4V5 prokaryotic reads and 18SV4 protistan reads

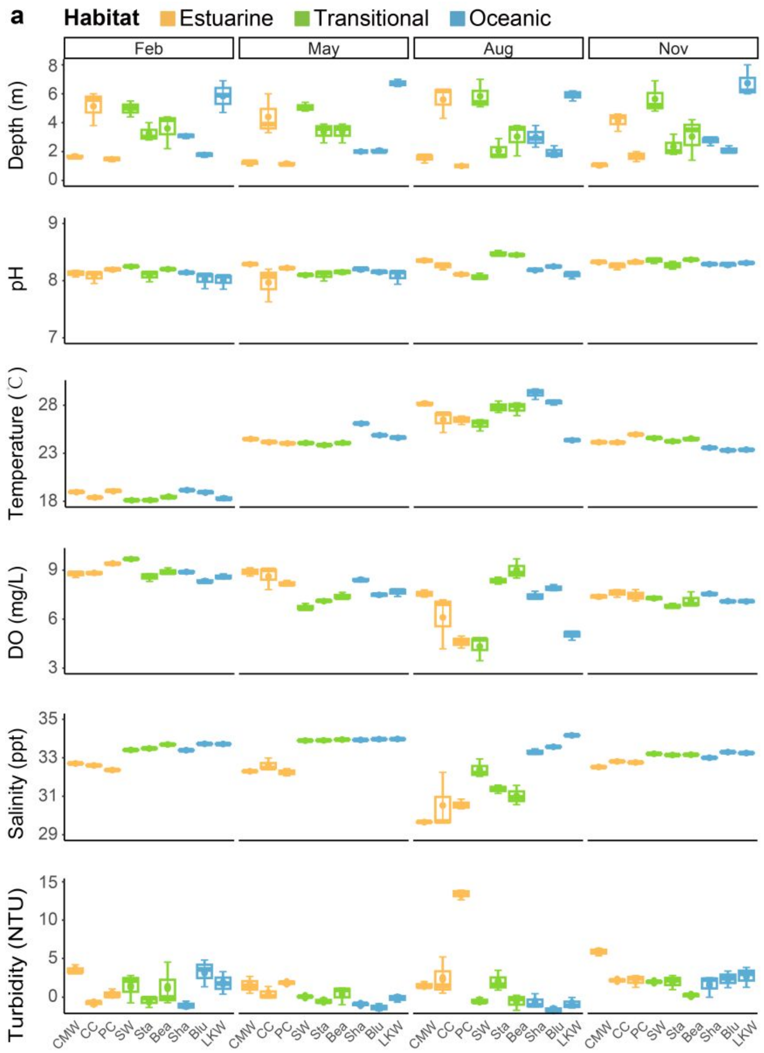
a
Habitat
Estuarine
Transitional
Oceanic
Feb
May
Aug
Nov
Depth (m)
pH
Temperature (℃)
DO (mg/L)
Salinity (ppt)
Turbidity (NTU)
CMW
CC
PC
SW
Sta
Bea
Sha
Blu
LKW

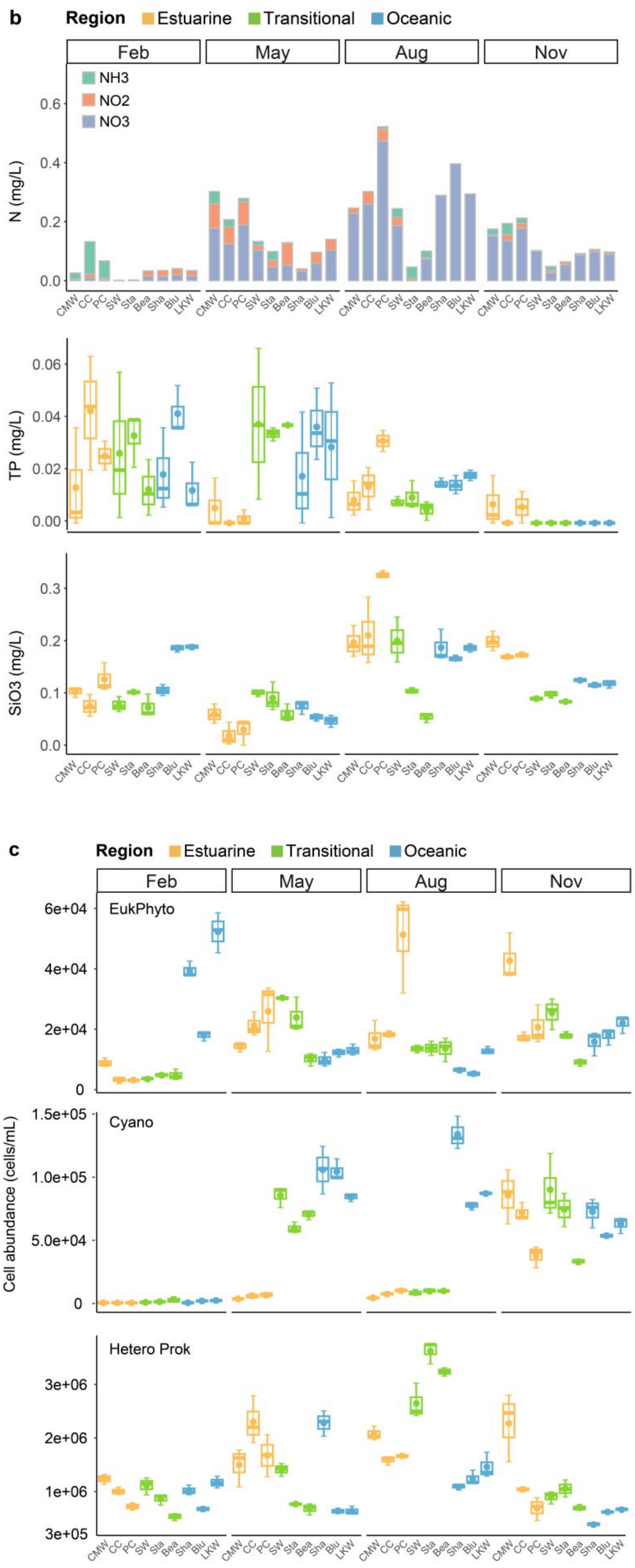

Figure S 9. Environmental factors and cell abundances across regions and seasons.
